# Supplementary material for: Self-carbon-thermal-reduction strategy for boosting the Fenton-like activity of single Fe-N4 sites by carbon-defect engineering
Source: Nat Commun. 2023 Nov 20;14:7549. doi: 10.1038/s41467-023-43040-5 (PMC10662205; doi:10.1038/s41467-023-43040-5)
Supplement: Supplementary file 1 — Supplementary Information [file 41467_2023_43040_MOESM1_ESM.pdf]

Self-carbon-thermal-reduction strategy for boosting the Fenton-like activity of single Fe-N_4 sites by carbon-defect engineering

Shengjie Wei[1, 2, 7] , Yibing Sun[3, 7], Yun-Ze Qiu[2, 7], Ang Li[4, 7], Ching-Yu Chiang[5*], Hai Xiao[2*], Jieshu Qian[3, 6*], Yadong Li[2*].

[1]School of Materials Science and Engineering, Nankai University, Tianjin 300350, China.

[2]Department of Chemistry, Tsinghua University, Beijing 100084, China.

[3]Jiangsu Key Laboratory of Chemical Pollution Control and Resources Reuse, School of Environmental and Biological Engineering, Nanjing University of Science and Technology, Nanjing, 210094, PR China.

[4]Faculty of Materials and Manufacturing, Beijing Key Lab of Microstructure and Properties of Advanced Materials, Beijing University of Technology, Beijing 100124, PR China.

[5]National Synchrotron Radiation Research Center, Hsinchu 30076, Taiwan.

[6]School of Environmental Engineering, Wuxi University, Jiangsu 214105, PR China.

[7]These authors contributed equally to this work.

[*]e-mail:

ydli@mail.tsinghua.edu.cn (Y.L.)

qianjieshu@njust.edu.cn (J.Q.)

haixiao@tsinghua.edu.cn (H.X.)

chiang.cy@nsrrc.org.tw (C.-Y.C.)

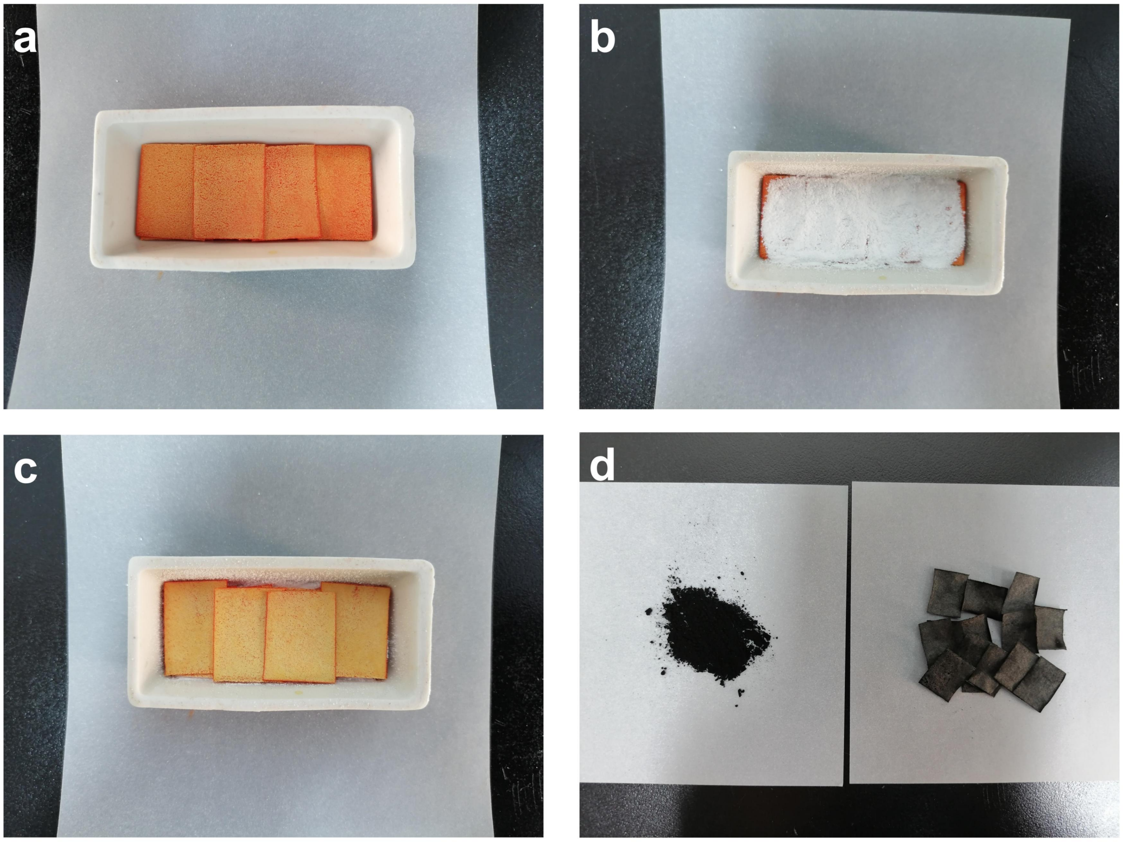

Supplementary Fig. 1. The photographs of the synthetic procedure of the Fe-NCv-900. **a**, The $Fe(acac)_3$@filter papers evenly paved the bottom of a ceramic boat. **b**, ZIF-8 powder homogeneously paved the $Fe(acac)_3$@filter papers. **c**, The $Fe(acac)_3$@filter papers evenly paved the ZIF-8 powder to form sandwich-like structure as the precursor of Fe-NCv-900. **d**, After pyrolysis at 900°C for 3h, Fe-NCv-900 was easily separated with the carbonized filter papers.

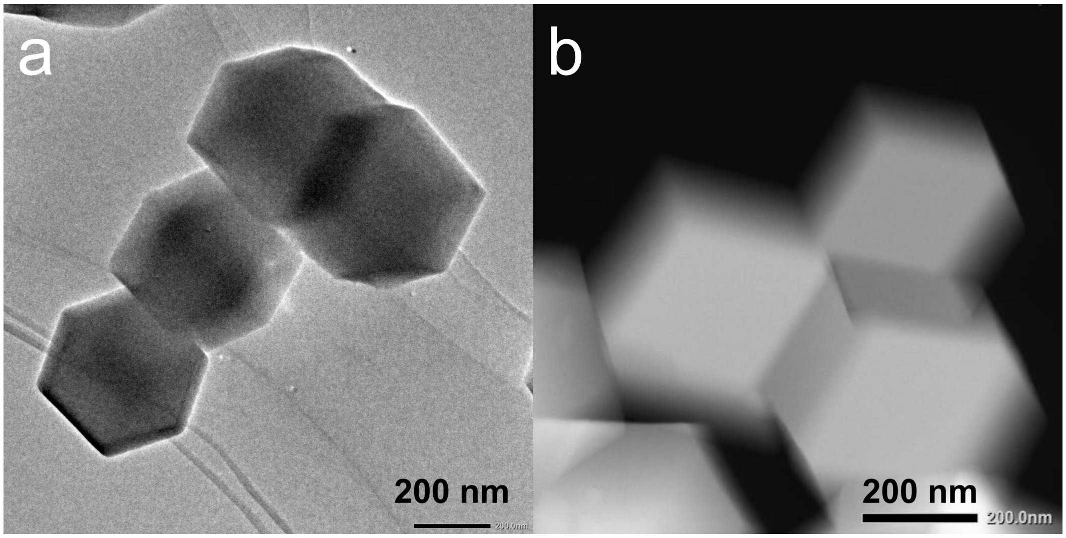

Supplementary Fig. 2. a The TEM image of ZIF-8. **b** The HAADF-STEM image of ZIF-8.

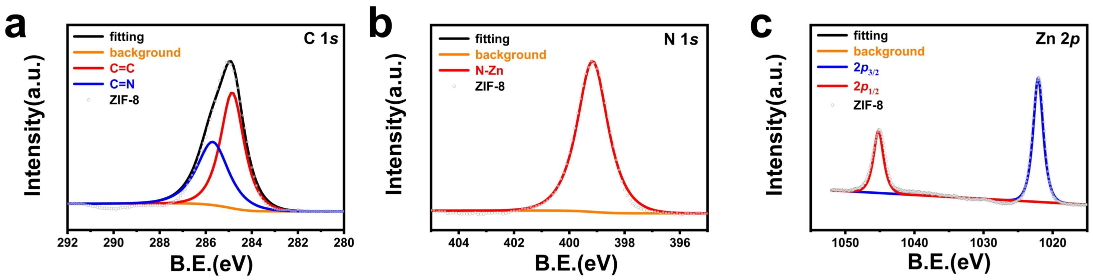

Supplementary Fig. 3. The XPS measurement and analysis of ZIF-8. **a**, XPS spectrum for the C 1*s*. **b**, XPS spectrum for the N 1*s*. **c**, XPS spectrum for the Zn 2*p*.

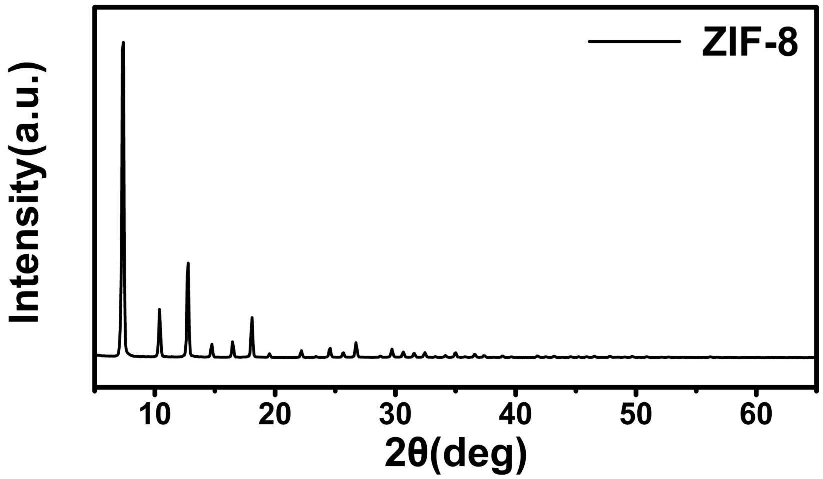

Supplementary Fig. 4. The XRD pattern of ZIF-8. The sharp diffraction peaks of ZIF-8 demonstrated the good crystallinity of ZIF-8.

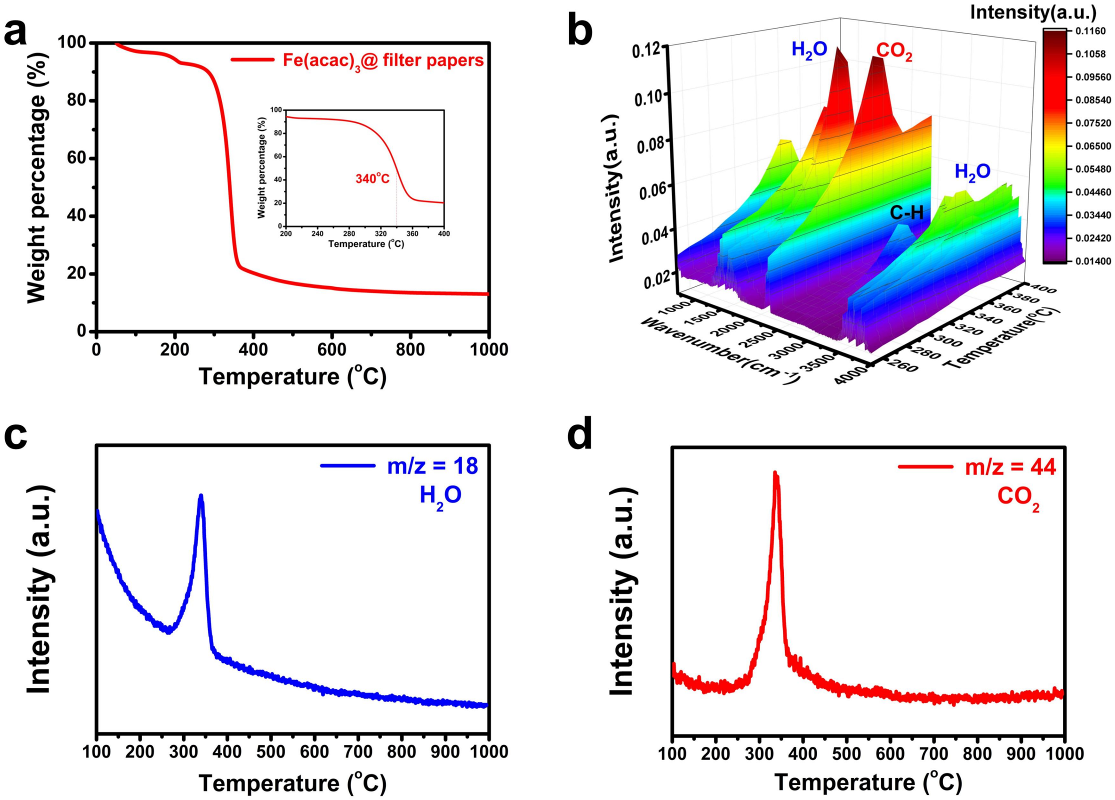

Supplementary Fig. 5. The thermogravimetric analysis coupled with fourier-transform infrared spectroscopy and mass spectrometry (TG-FTIR-MS) measurements of the released gases during pyrolysis of Fe(acac)$_3$@filter papers from room temperature to 1000°C (heating rate: 5°C/min, argon atmosphere). **a**, The thermogravimetric analysis of Fe(acac)$_3$@filter papers. **b**, The TG-FTIR spectrum of the released gases during pyrolysis from 250°C to 400°C. The two broad peaks around 1600 cm^{-1} and 3600 cm^{-1} were ascribed to the H_2O vapour (*ACS Catal.* **12**, 10771-10780 (2022).) due to the decomposition of the hydroxyl functional group from the cellulose of filter papers. While the peak around 2360 cm^{-1} was ascribed to the CO_2 gas (*Chem* **7**, 1297-1307 (2021).) **c** and **d**, the TG-MS spectra of the released H_2O (m/z = 18) and CO_2 (m/z = 44) gases, respectively. The Fe(acac)$_3$@filter papers released the H_2O and CO_2 gases from 250°C to 400°C, and the maximum intensity of signal was around 340°C.

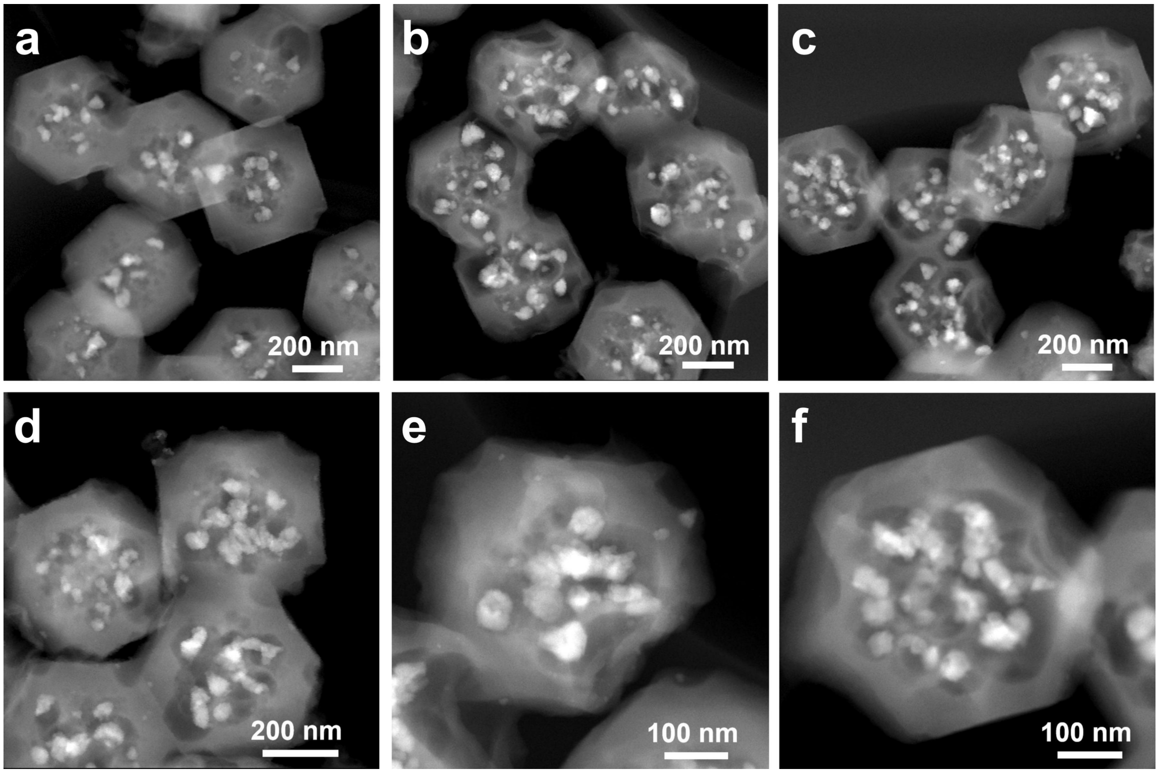

Supplementary Fig. 6. a-**f**, The HAADF-STEM images of the Fe-NCv-400 catalyst from different regions at different magnifications. The in situ released H_2O vapor from filter papers reacted with the Zn^{2+} ions from ZIF-8 and ZnO NPs as nano-oxidant were in situ formed within ZIF-8. The nano-reactor (ZnO NPs as nano-oxidant and carbon-substrate as nano-reductant) was in situ constructed as the precursors during the subsequent self-carbon-thermal-reduction.

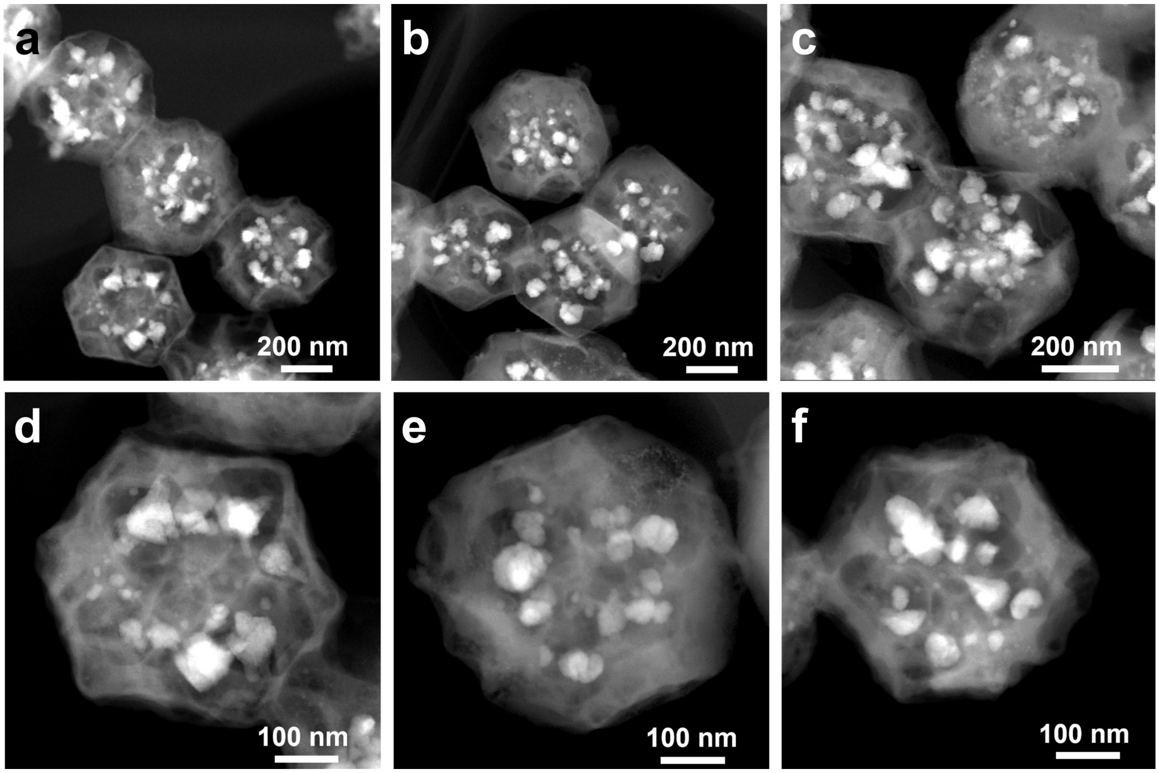

Supplementary Fig. 7. a-f, The HAADF-STEM images of the Fe-NCv-500 catalyst from different regions at different magnifications. The ZIF-8-derived CN substrate was carbonized at 500°C with ZnO NPs remaining.

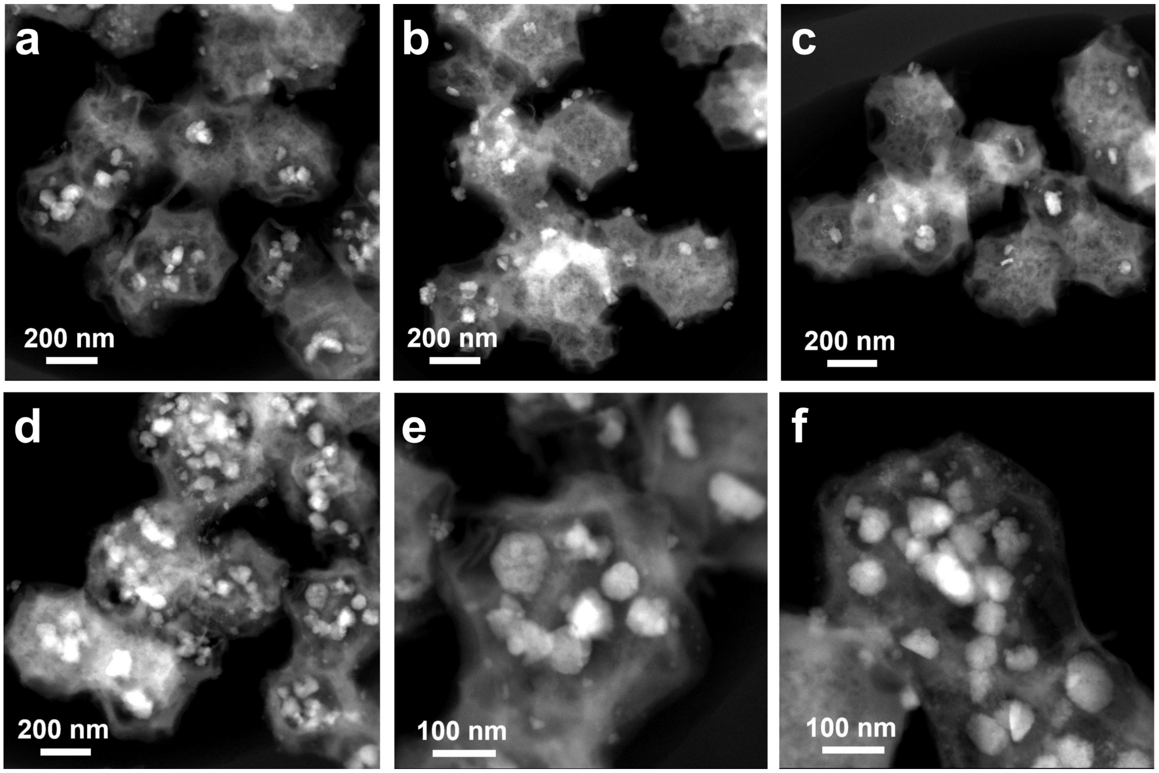

Supplementary Fig. 8. a-f, The HAADF-STEM images of the Fe-NCv-600 catalyst from different regions at different magnifications. The ZIF-8-derived CN substrate was further carbonized at 600°C with ZnO NPs remaining.

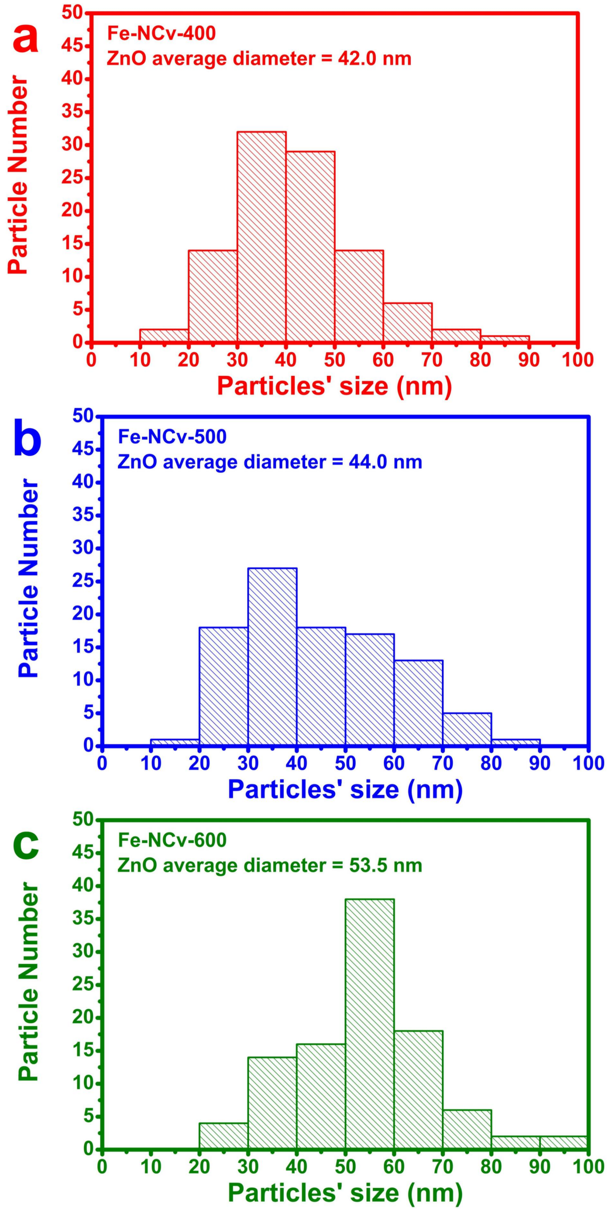

Supplementary Fig. 9. a-c, The particle size distribution of 100 ZnO NPs in Fe-NCv-400, Fe-NCv-500 and Fe-NCv-600, respectively, and corresponding average diameters of ZnO NPs. From 400°C to 600°C, the ZnO NPs in situ formed and grew larger, with the average diameters of ZnO NPs increasing from 42.0 nm, 44.0 nm and 53.5 nm at 400°C, 500°C and 600°C, respectively.

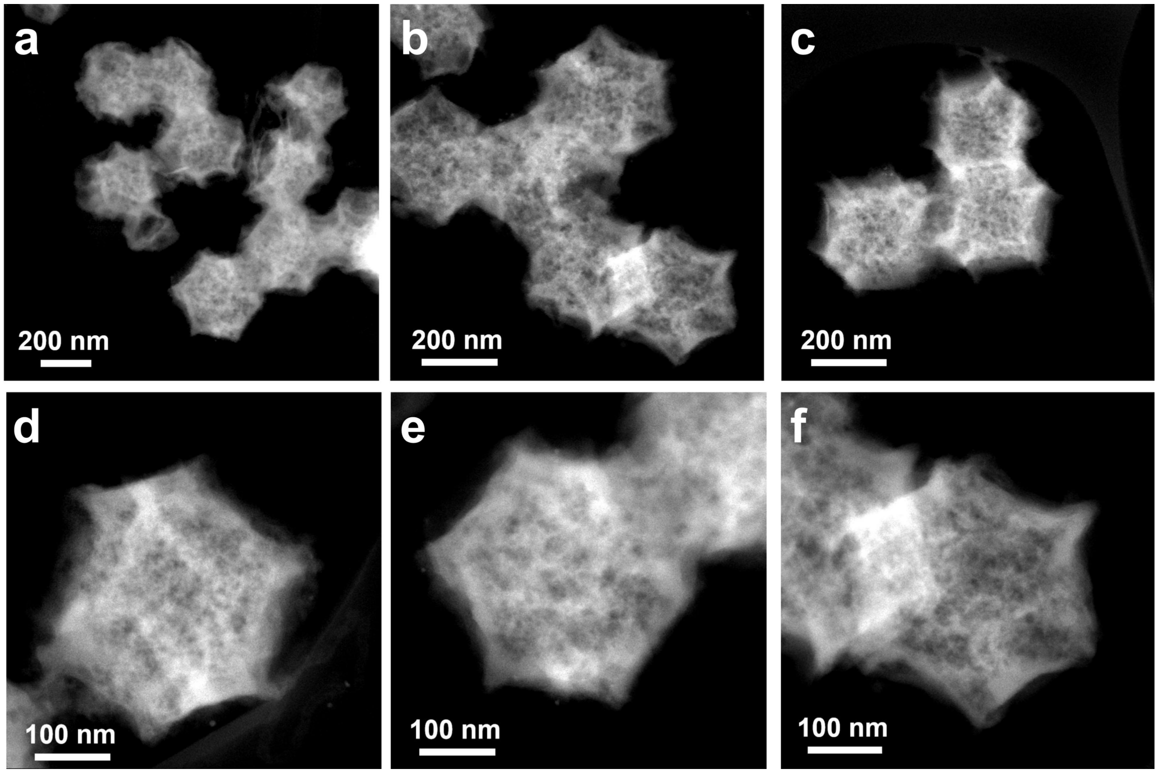

Supplementary Fig. 10. a-f, The HAADF-STEM images of the Fe-NCv-700 catalyst from different regions at different magnifications. The self-carbon-thermal-reduction occurred spontaneously in ZnO-carbon nano-reactor (ZnO NPs as nano-oxidant and carbon-substrate as nano-reductant), with in situ disappearance of ZnO NPs and formation of carbon-defects.

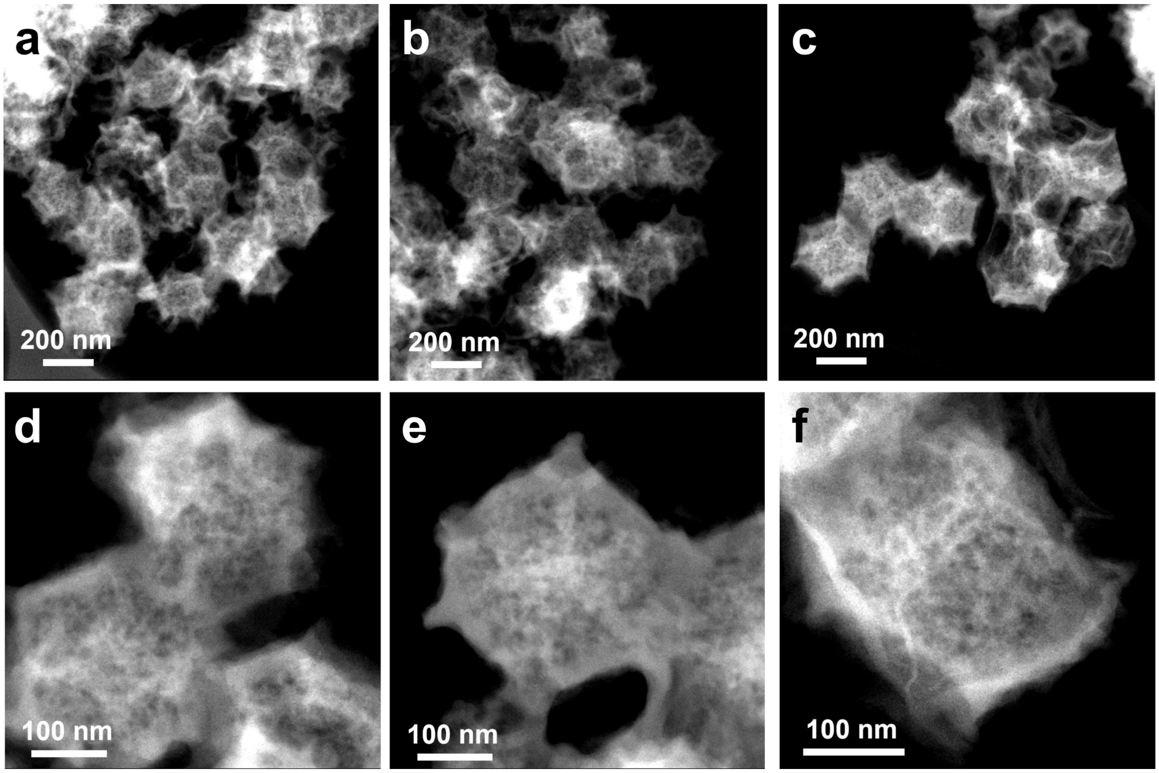

Supplementary Fig. 11. a-f, The HAADF-STEM images of the Fe-NCv-800 catalyst from different regions at different magnifications. No ZnO nanoparticles existed in Fe-NCv-800 catalyst by self-carbon-thermal-reduction with the formation of mesopores and macropores.

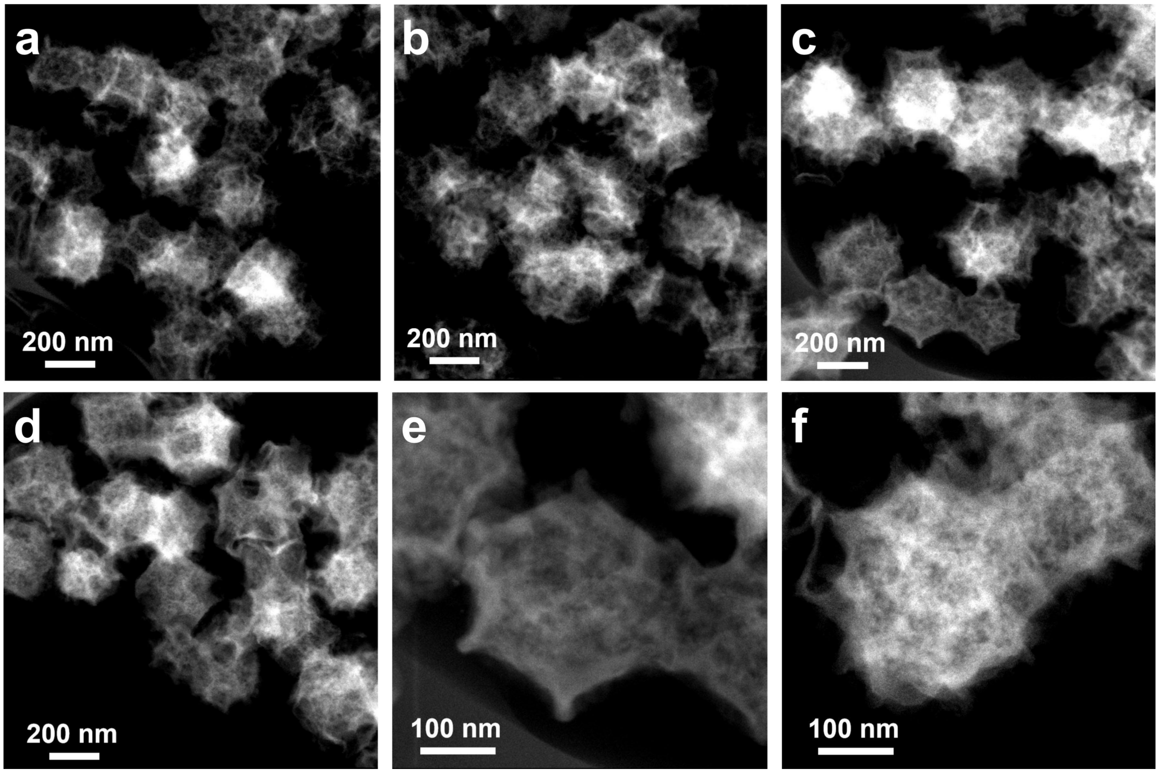

Supplementary Fig. 12. a-f, The HAADF-STEM images of the Fe-NCv-900 catalyst from different regions at different magnifications. No ZnO nanoparticles was found in Fe-NCv-900 catalyst by self-carbon-thermal-reduction with the formation of mesopores and macropores.

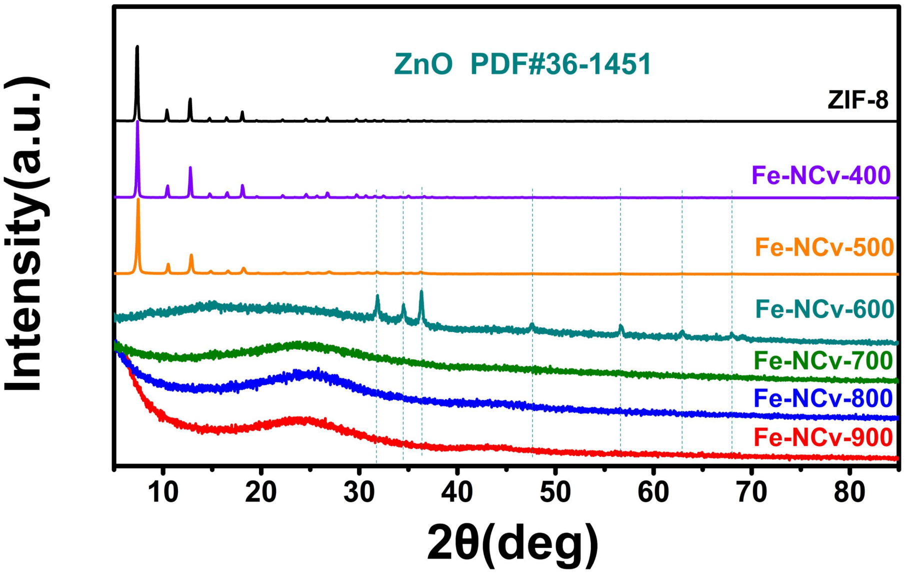

Supplementary Fig. 13. The XRD patterns of ZIF-8, Fe-NCv-400, Fe-NCv-500, Fe-NCv-600, Fe-NCv-700, Fe-NCv-800, and Fe-NCv-900. ZIF-8 was carbonized above 600°C, with the vanishment of diffraction peaks of ZIF-8. The XRD diffraction peaks of ZnO (PDF#36-1451) existed in Fe-NCv-500 and Fe-NCv-600 while those in situ disappeared in Fe-NCv-700, Fe-NCv-800 and Fe-NCv-900, confirming the in situ formation and in situ disappearance of ZnO NPs. The Fe-NCv-900 only had two broad peaks around 25° and 44°, which was ascribed to the characteristic carbon (002) and (100)/(101) diffractions (*Nature Nanotech.* **13**, 856-861 (2018).) and no peaks of Fe particles or ferric oxide could be found.

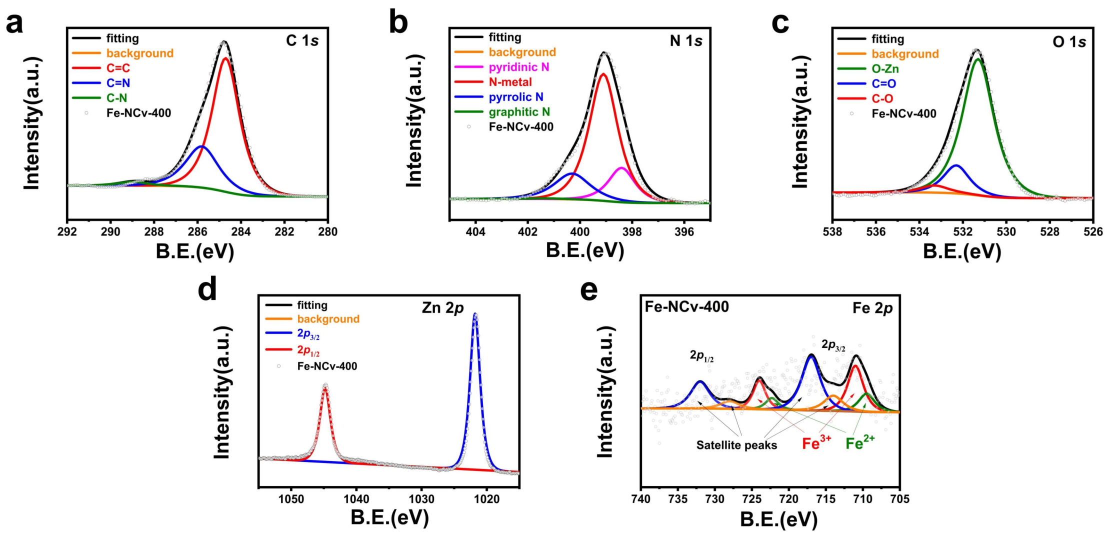

Supplementary Fig. 14. The XPS spectra of Fe-NCv-400. **a**, XPS spectrum for the C 1*s*. **b**, XPS spectrum for the N 1*s*. **c**, XPS spectrum for the O 1*s*. **d**, XPS spectrum for the Zn 2*p*. **e**, XPS spectrum for the Fe 2*p*.

For C 1*s* spectra, C=C, C=N and C-N bonds (*Angew. Chem. Int. Ed.* **59**, 22465-22469 (2020).) co-existed.

For N 1*s* spectra, pyridinic N, N-metal, pyrrolic N, and graphitic N species (*Angew. Chem. Int. Ed.* **60**, 9078-9085 (2021).) co-existed.

For O 1*s* spectra, C=O, C-O bonds (*Angew. Chem. Int. Ed.* **59**, 1961-1965 (2020).) and O-Zn bond (*Appl. Catal. B-Environ.* **310**, 121298 (2022).) co-existed.

We referred the reference (*Nat. Mater.* **20**, 1385-1391 (2021).) to analyze the Fe^{3+}, Fe^{2+} components and the satellite peaks.

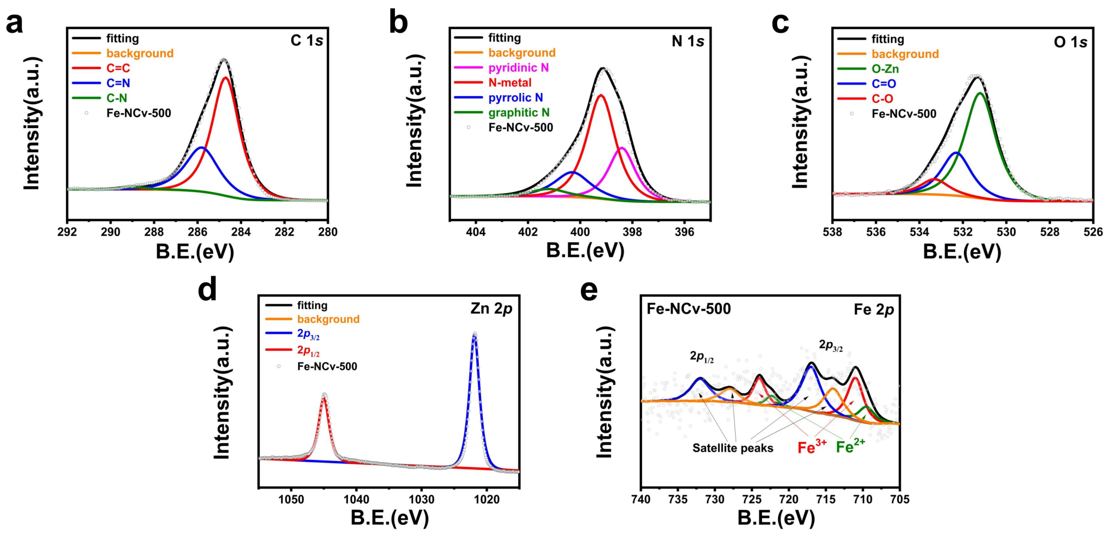

Supplementary Fig. 15. The XPS spectra of Fe-NCv-500. **a**, XPS spectrum for the C 1*s*. **b**, XPS spectrum for the N 1*s*. **c**, XPS spectrum for the O 1*s*. **d**, XPS spectrum for the Zn 2*p*. **e**, XPS spectrum for the Fe 2*p*.

For C 1*s* spectra, C=C, C=N and C-N bonds (*Angew. Chem. Int. Ed.* **59**, 22465-22469 (2020).) co-existed.

For N 1*s* spectra, pyridinic N, N-metal, pyrrolic N, and graphitic N species (*Angew. Chem. Int. Ed.* **60**, 9078-9085 (2021).) co-existed.

For O 1*s* spectra, C=O, C-O bonds (*Angew. Chem. Int. Ed.* **59**, 1961-1965 (2020).) and O-Zn bond (*Appl. Catal. B-Environ.* **310**, 121298 (2022).) co-existed.

We referred the reference (*Nat. Mater.* **20**, 1385-1391 (2021).) to analyze the Fe^{3+}, Fe^{2+} components and the satellite peaks.

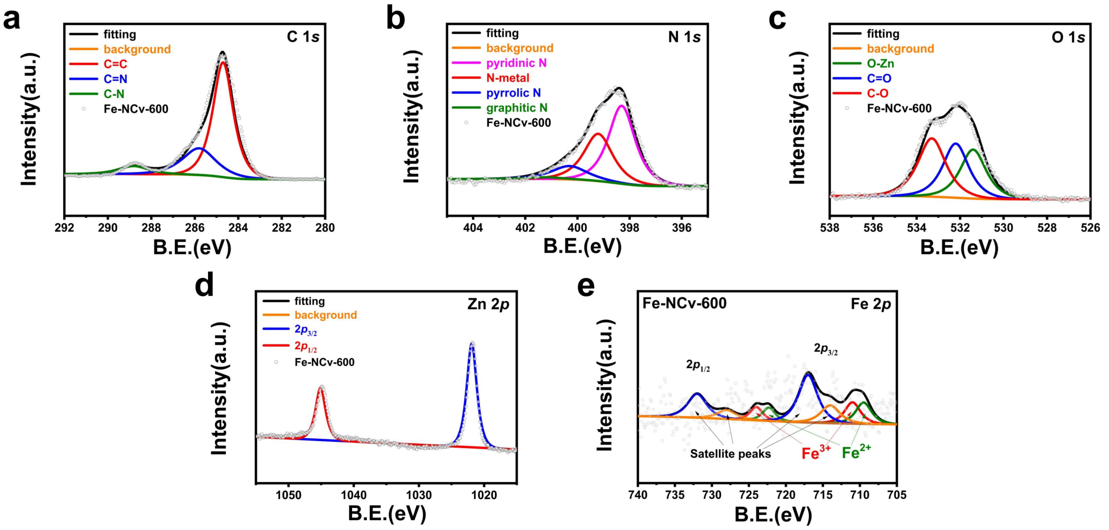

Supplementary Fig. 16. The XPS spectra of Fe-NCv-600. **a**, XPS spectrum for the C 1*s*. **b**, XPS spectrum for the N 1*s*. **c**, XPS spectrum for the O 1*s*. **d**, XPS spectrum for the Zn 2*p*. **e**, XPS spectrum for the Fe 2*p*.

For C 1*s* spectra, C=C, C=N and C-N bonds (*Angew. Chem. Int. Ed.* **59**, 22465-22469 (2020).) co-existed.

For N 1*s* spectra, pyridinic N, N-metal, pyrrolic N, and graphitic N species (*Angew. Chem. Int. Ed.* **60**, 9078-9085 (2021).) co-existed.

For O 1*s* spectra, C=O, C-O bonds (*Angew. Chem. Int. Ed.* **59**, 1961-1965 (2020).) and O-Zn bond (*Appl. Catal. B-Environ.* **310**, 121298 (2022).) co-existed.

We referred the reference (*Nat. Mater.* **20**, 1385-1391 (2021).) to analyze the Fe^{3+}, Fe^{2+} components and the satellite peaks.

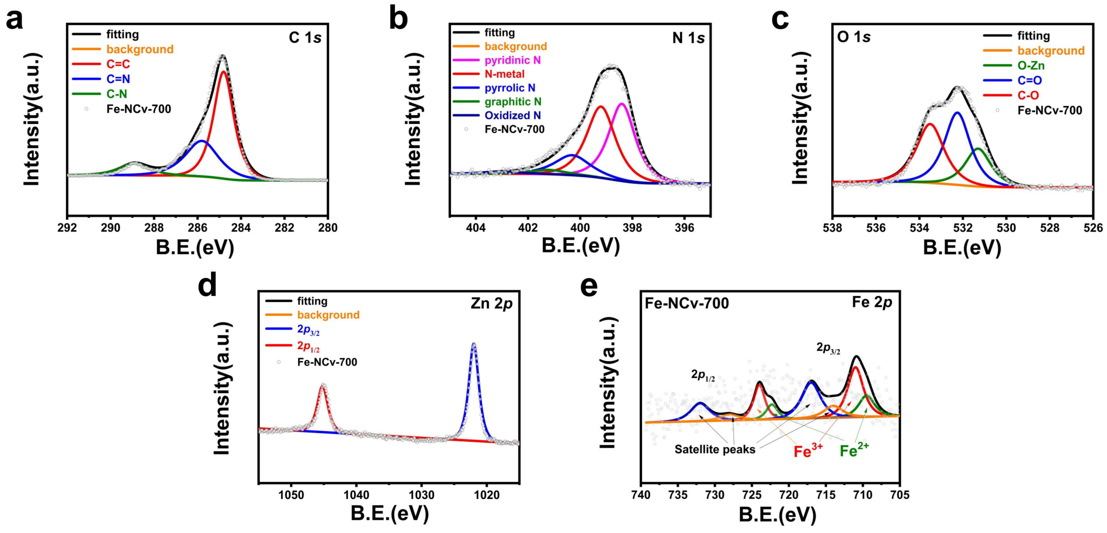

Supplementary Fig. 17. The XPS spectra of Fe-NCv-700. **a**, XPS spectrum for the C 1*s*. **b**, XPS spectrum for the N 1*s*. **c**, XPS spectrum for the O 1*s*. **d**, XPS spectrum for the Zn 2*p*. **e**, XPS spectrum for the Fe 2*p*.

For C 1*s* spectra, C=C, C=N and C-N bonds (*Angew. Chem. Int. Ed.* **59**, 22465-22469 (2020).) co-existed.

For N 1*s* spectra, pyridinic N, N-metal, pyrrolic N, graphitic N and oxidized N species (*Angew. Chem. Int. Ed.* **60**, 9078-9085 (2021).) co-existed.

For O 1*s* spectra, C=O, C-O bonds (*Angew. Chem. Int. Ed.* **59**, 1961-1965 (2020).) and O-Zn bond (*Appl. Catal. B-Environ.* **310**, 121298 (2022).) co-existed.

We referred the reference (*Nat. Mater.* **20**, 1385-1391 (2021).) to analyze the Fe^{3+}, Fe^{2+} components and the satellite peaks.

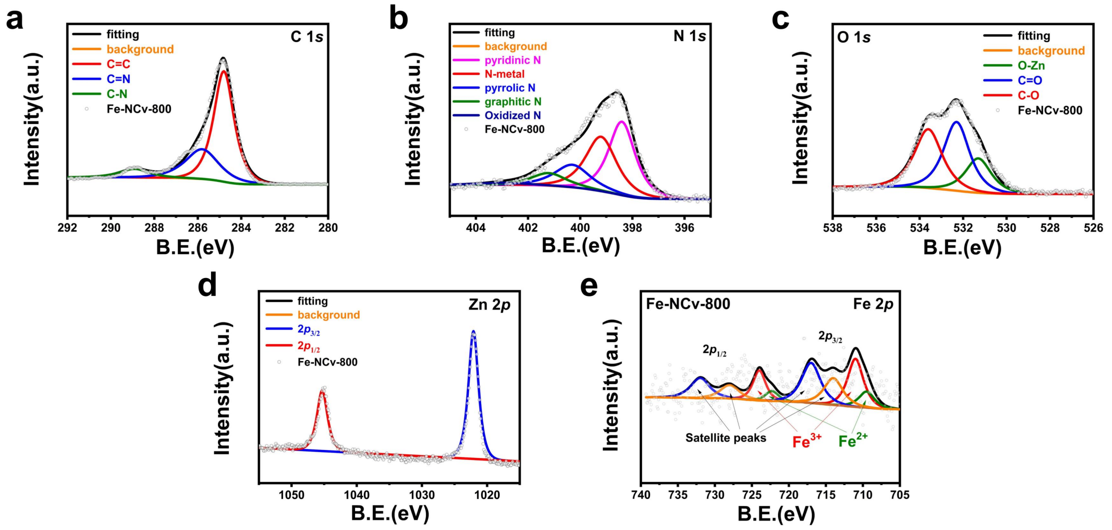

Supplementary Fig. 18. The XPS spectra of Fe-NCv-800. **a**, XPS spectrum for the C 1*s*. **b**, XPS spectrum for the N 1*s*. **c**, XPS spectrum for the O 1*s*. **d**, XPS spectrum for the Zn 2*p*. **e**, XPS spectrum for the Fe 2*p*.

For C 1*s* spectra, C=C, C=N and C-N bonds (*Angew. Chem. Int. Ed.* **59**, 22465-22469 (2020).) co-existed.

For N 1*s* spectra, pyridinic N, N-metal, pyrrolic N, graphitic N and oxidized N species (*Angew. Chem. Int. Ed.* **60**, 9078-9085 (2021).) co-existed.

For O 1*s* spectra, C=O, C-O bonds (*Angew. Chem. Int. Ed.* **59**, 1961-1965 (2020).) and O-Zn bond (*Appl. Catal. B-Environ.* **310**, 121298 (2022).) co-existed.

We referred the reference (*Nat. Mater.* **20**, 1385-1391 (2021).) to analyze the Fe^{3+}, Fe^{2+} components and the satellite peaks.

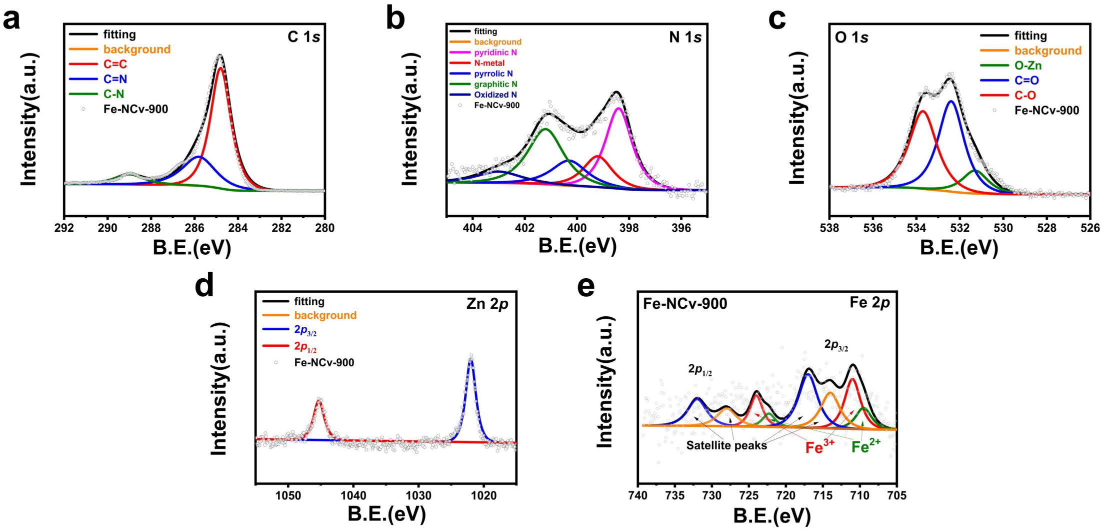

Supplementary Fig. 19. The XPS spectra of Fe-NCv-900. **a**, XPS spectrum for the C 1*s*. **b**, XPS spectrum for the N 1*s*. **c**, XPS spectrum for the O 1*s*. **d**, XPS spectrum for the Zn 2*p*. **e**, XPS spectrum for the Fe 2*p*.

For C 1*s* spectra, C=C, C=N and C-N bonds (*Angew. Chem. Int. Ed.* **59**, 22465-22469 (2020).) co-existed.

For N 1*s* spectra, pyridinic N, N-metal, pyrrolic N, graphitic N and oxidized N species (*Angew. Chem. Int. Ed.* **60**, 9078-9085 (2021).) co-existed.

For O 1*s* spectra, C=O, C-O bonds (*Angew. Chem. Int. Ed.* **59**, 1961-1965 (2020).) and O-Zn bond (*Appl. Catal. B-Environ.* **310**, 121298 (2022).) co-existed.

We referred the reference (*Nat. Mater.* **20**, 1385-1391 (2021).) to analyze the Fe^{3+}, Fe^{2+} components and the satellite peaks.

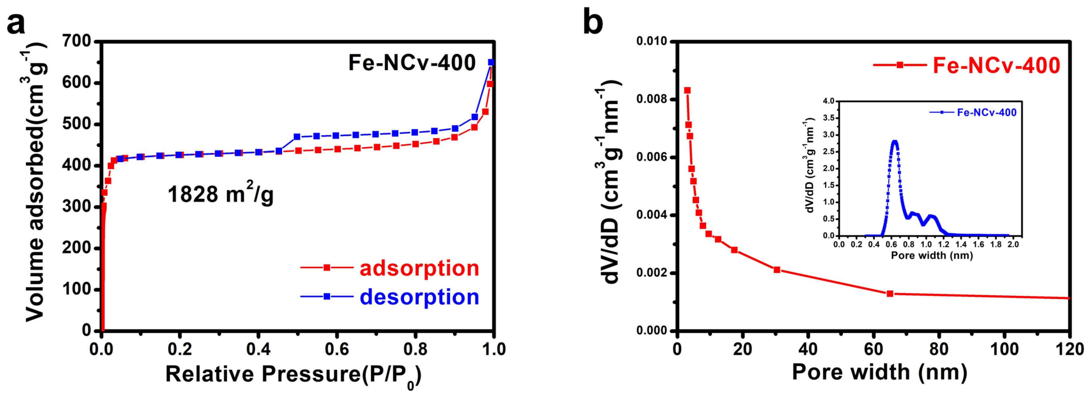

Supplementary Fig. 20. The BET surface area and pore size distribution of Fe-NCv-400. **a**, N_2 adsorption-desorption isotherms and corresponding BET surface area. **b**, The mesopore-size distribution and micropore-size distribution (insert) of Fe-NCv-400.

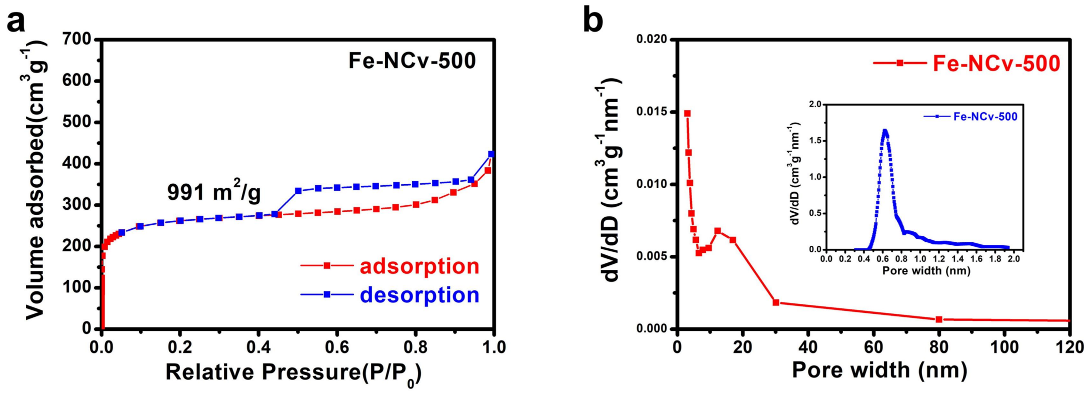

Supplementary Fig. 21. The BET surface area and pore size distribution of Fe-NCv-500. **a**, N_2 adsorption-desorption isotherms and corresponding BET surface area. **b**, The mesopore-size distribution and micropore-size distribution (insert) of Fe-NCv-500.

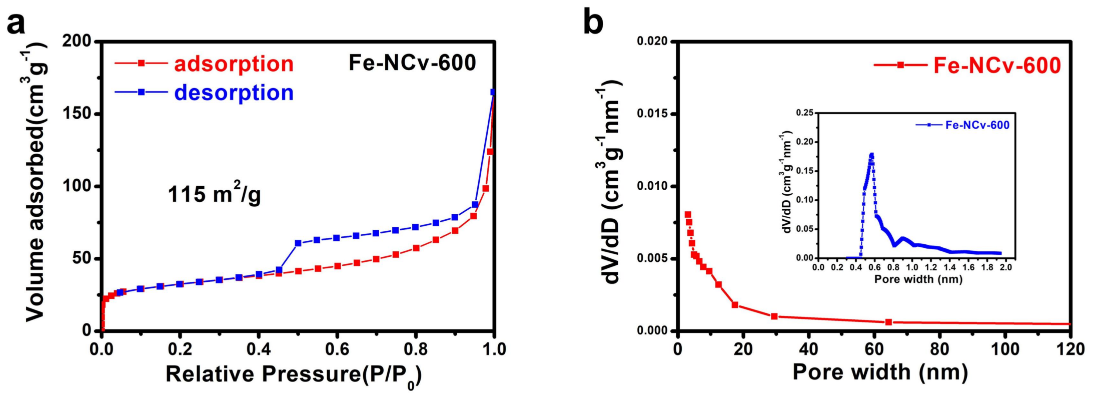

Supplementary Fig. 22. The BET surface area and pore size distribution of Fe-NCv-600. **a**, N_2 adsorption-desorption isotherms and corresponding BET surface area. **b**, The mesopore-size distribution and micropore-size distribution (insert) of Fe-NCv-600.

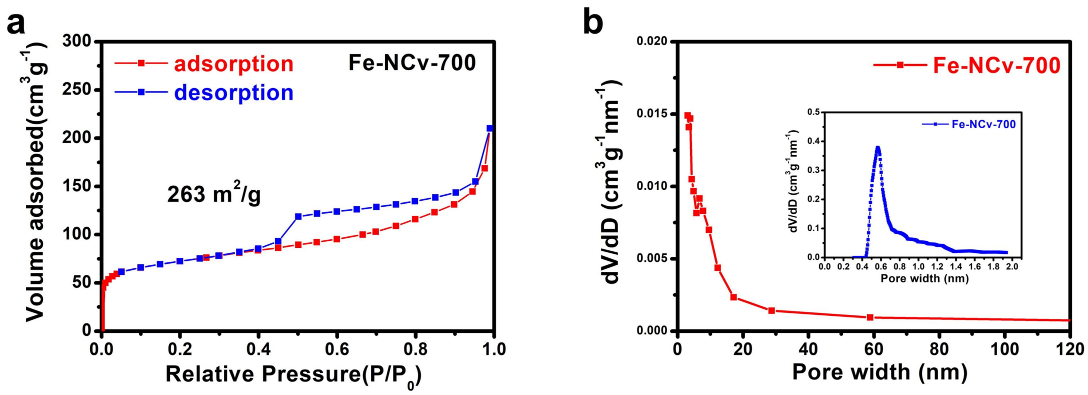

Supplementary Fig. 23. The BET surface area and pore size distribution of Fe-NCv-700. **a**, N_2 adsorption-desorption isotherms and corresponding BET surface area. **b**, The mesopore-size distribution and micropore-size distribution (insert) of Fe-NCv-700.

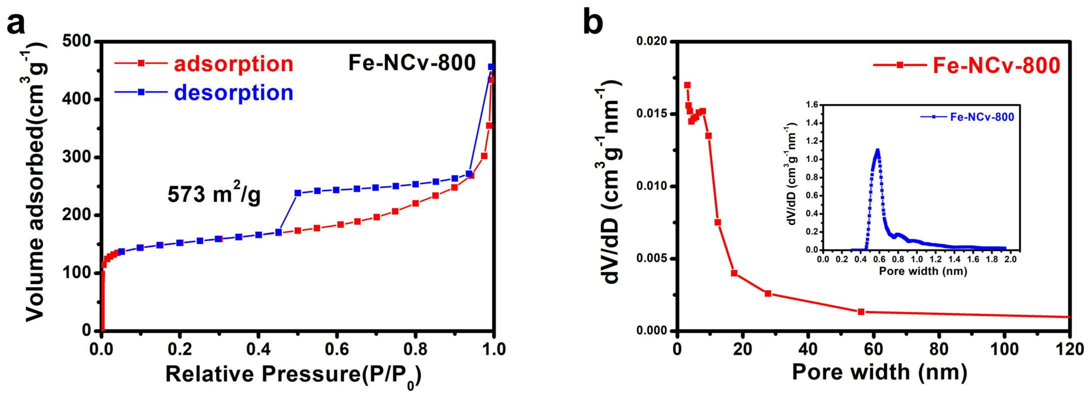

Supplementary Fig. 24. The BET surface area and pore size distribution of Fe-NCv-800. **a**, N_2 adsorption-desorption isotherms and corresponding BET surface area. **b**, The mesopore-size distribution and micropore-size distribution (insert) of Fe-NCv-800.

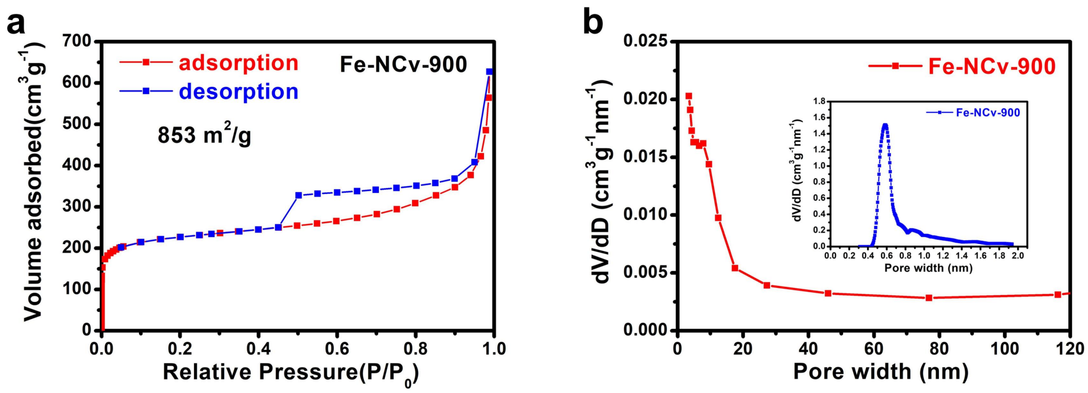

Supplementary Fig. 25. The BET surface area and pore size distribution of Fe-NCv-900. **a**, N_2 adsorption-desorption isotherms and corresponding BET surface area. **b**, The mesopore-size distribution and micropore-size distribution (insert) of Fe-NCv-900.

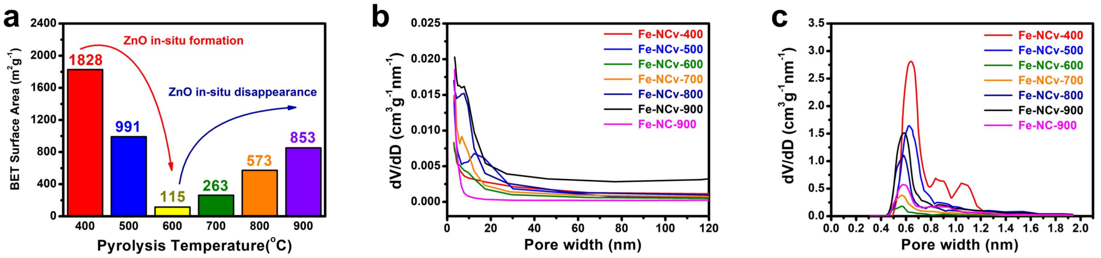

Supplementary Fig. 26. The comparison of BET surface area and mesopore's size distribution of Fe-NCv samples. **a**, the summary of BET surface area of Fe-NCv samples. During in situ formation of ZnO NPs and collapse of ZIF-8, the BET surface areas decreased sharply from 1828 m^2/g of Fe-NCv-400, to 991 m^2/g of Fe-NCv-500, and to 115 m^2/g of Fe-NCv-600. For in situ disappearance of ZnO NPs during self-carbon-thermal reduction, the BET surface areas increased gradually from 263 m^2/g of Fe-NCv-700, to 573 m^2/g of Fe-NCv-800, and 853 m^2/g of Fe-NCv-900. **b**, the summary of mesopore's size distribution of Fe-NCv samples. **c**, the summary of micropore's size distribution of Fe-NCv samples.

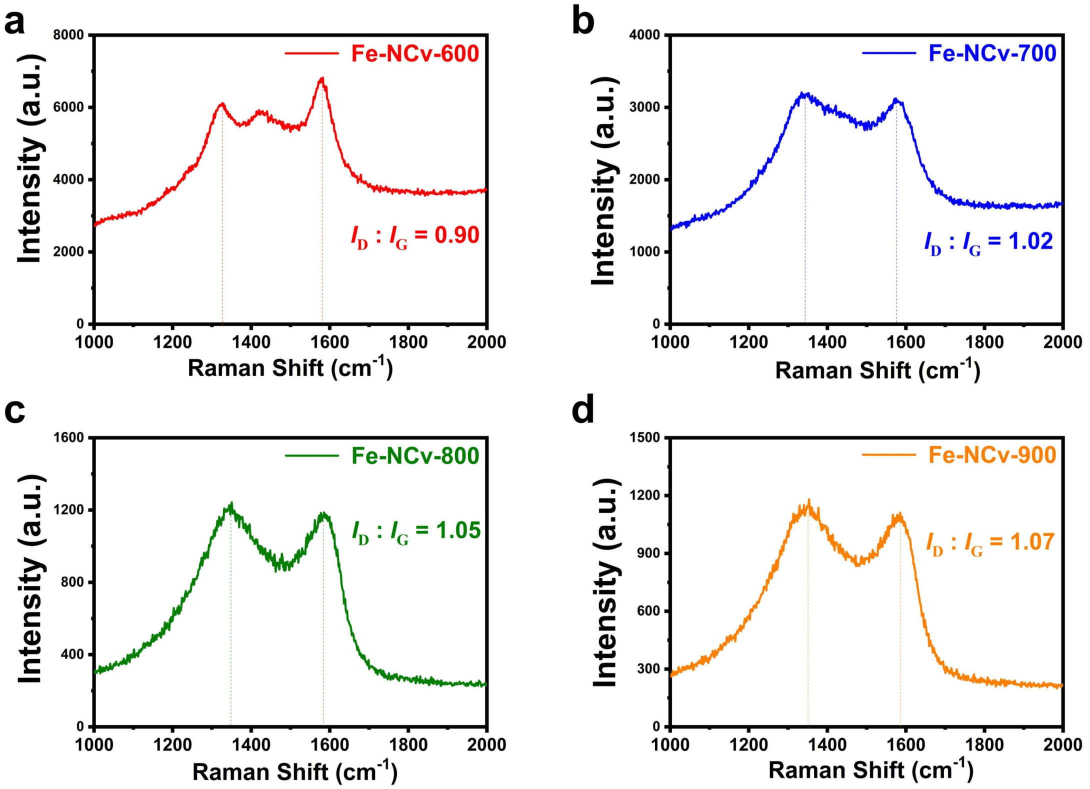

Supplementary Fig. 27. a-d, The Raman spectra of Fe-NCv-600, Fe-NCv-700, Fe-NCv-800 and Fe-NCv-900, respectively.

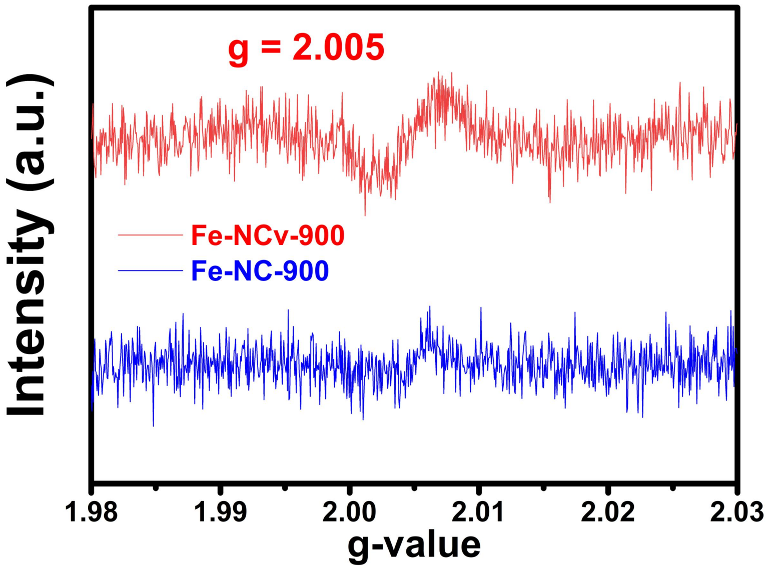

Supplementary Fig. 28. The EPR spectra of Fe-NCv-900 and Fe-NC-900. There was an obvious signal at the g-value of 2.005 g in the EPR pattern of Fe-NCv-900, revealing the existence of carbon-vacancies in the carbon substrate of Fe-NCv-900.

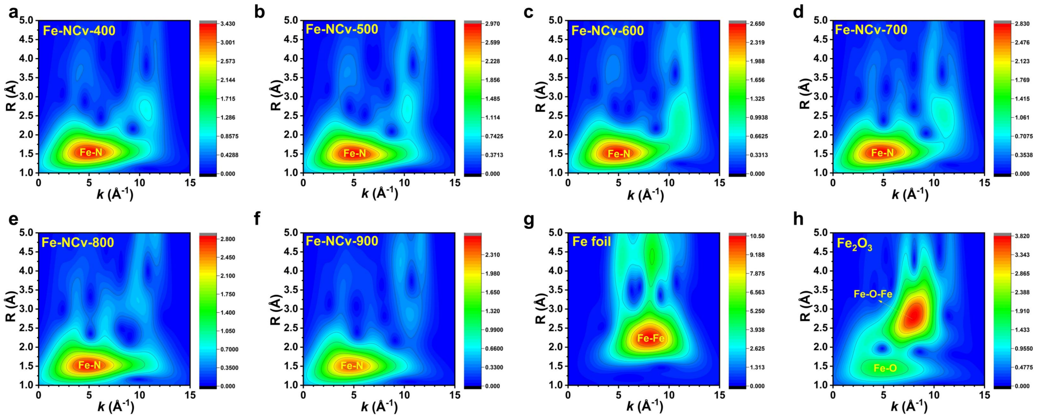

Supplementary Fig. 29. a-h, The WT analysis at Fe K-edge of Fe-NCv-400, Fe-NCv-500, Fe-NCv-600, Fe-NCv-700, Fe-NCv-800, Fe-NCv-900, Fe foil and Fe_2O_3 as reference samples. We compare the contour plots of Fe-NCv catalysts, Fe_2O_3 and Fe foil as reference samples. In **Supplementary Fig. 29g**, the dominant peak at around 8.0 $Å^{-1}$ in k space and 2.2 Å in R space of Fe foil is ascribed to Fe-Fe bond. In **Supplementary Fig. 29h**, the dominant peak at around 8.5 $Å^{-1}$ in k space and 3.0 Å in R space of Fe_2O_3 is ascribed to Fe-O-Fe bond while the secondary dominant peak at around 5.0 $Å^{-1}$ in k space and 1.5 Å in R space of Fe_2O_3 is ascribed to Fe-O bond. By comparison, all the Fe-NCv catalysts only have one prominent peak at around 5.0 $Å^{-1}$ in k space and 1.5 Å in R space assigning to Fe-N bond, without the characteristic peaks of Fe-Fe bond or Fe-O-Fe bond, indicating that the Fe element of the Fe-NCv catalysts exists as isolated single-atom sites. During the in situ formation of ZnO nanoparticles, the in situ released water vapor from filter papers reacts with Zn^{2+} ions from ZIF-8, with the breaking of Zn-N bonds and agglomeration of Zn element. The coordination-unsaturated N atoms from the broken Zn-N bonds serve as the anchoring sites of Fe species, with the formation of Fe-ISAS. During the in situ disappearance of ZnO nanoparticles by self-carbon-thermal-reduction in ZnO-Carbon nano-reactor, the carbon vacancies are introduced around Fe-ISAS, which effectively boost the catalytic activity of Fe-ISAS for Fenton-like reactions. Therefore, the in situ formation of ZnO nanoparticles facilitates the formation of Fe-ISAS and the in

situ disappearance of ZnO nanoparticles induces the formation of carbon vacancies around Fe ISAS.

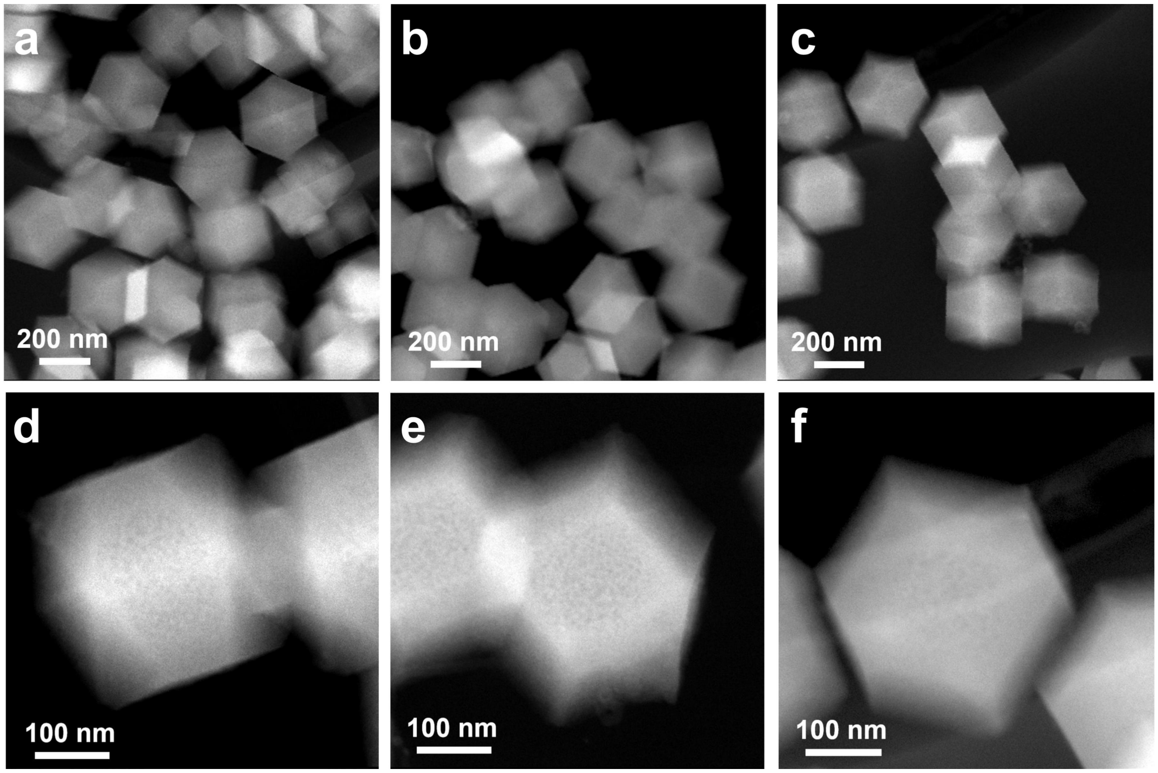

Supplementary Fig. 30. a-f, The HAADF-STEM images of the Fe-NC-900 catalyst from different regions at different magnifications. The Fe-NC-900 catalyst was obtained by normal pyrolysis of $Fe(acac)_3$@ZIF-8, without formation of abundant carbon-defect.

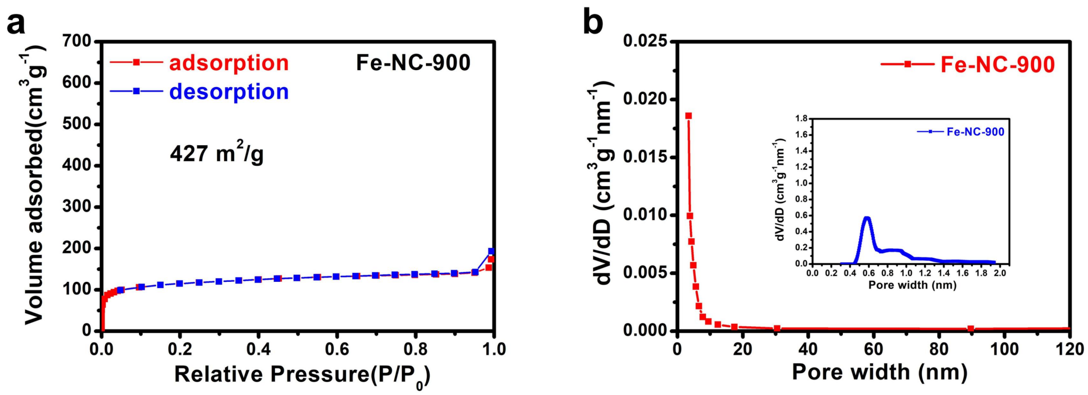

Supplementary Fig. 31. The BET surface area and pore size distribution of Fe-NC-900. **a**, N_2 adsorption-desorption isotherms and corresponding BET surface area. **b**, The mesopore-size distribution and micropore-size distribution (insert) of Fe-NC-900.

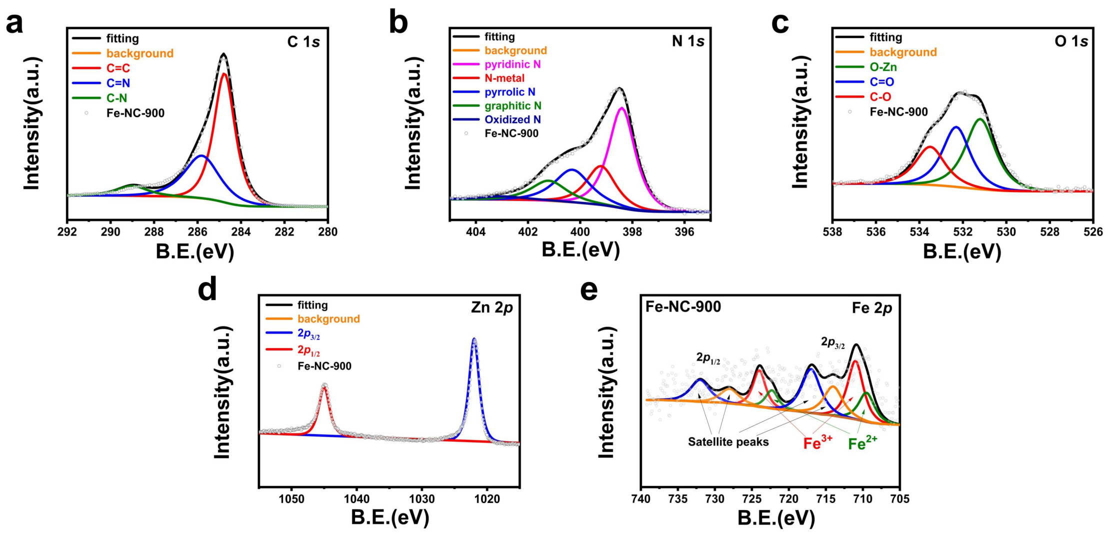

Supplementary Fig. 32. The XPS spectra of Fe-NC-900. **a**, XPS spectrum for the C 1*s*. **b**, XPS spectrum for the N 1*s*. **c**, XPS spectrum for the O 1*s*. **d**, XPS spectrum for the Zn 2*p*. **e**, XPS spectrum for the Fe 2*p*.

For C 1*s* spectra, C=C, C=N and C-N bonds (*Angew. Chem. Int. Ed.* **59**, 22465-22469 (2020).) co-existed.

For N 1*s* spectra, pyridinic N, N-metal, pyrrolic N, graphitic N and oxidized N species (*Angew. Chem. Int. Ed.* **60**, 9078-9085 (2021).) co-existed.

For O 1*s* spectra, C=O, C-O bonds (*Angew. Chem. Int. Ed.* **59**, 1961-1965 (2020).) and O-Zn bond (*Appl. Catal. B-Environ.* **310**, 121298 (2022).) co-existed.

We referred the reference (*Nat. Mater.* **20**, 1385-1391 (2021).) to analyze the Fe^{3+}, Fe^{2+} components and the satellite peaks.

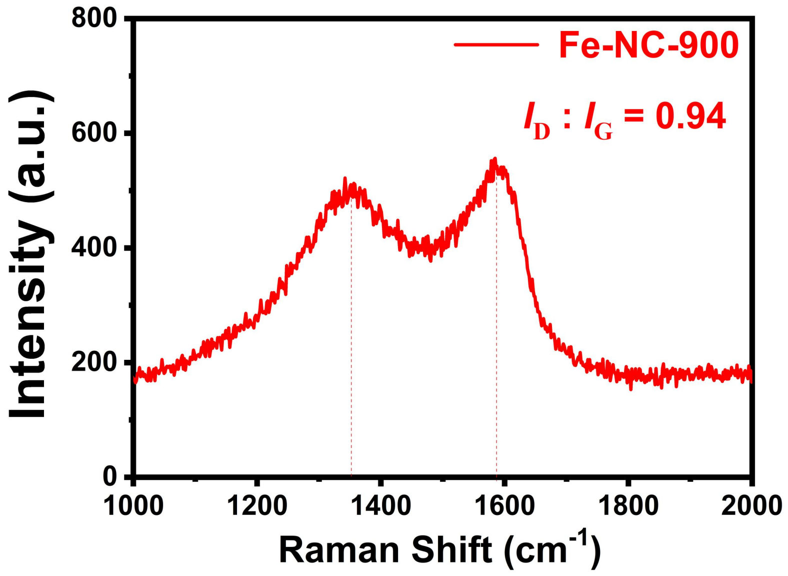

Supplementary Fig. 33. The Raman spectrum of Fe-NC-900. The I_D/I_G value of Fe-NC-900 catalyst was 0.94, lower than that of Fe-NCv-900 catalyst (I_D/I_G value of 1.07), indicating the larger degree of disorder from carbon substrates of Fe-NCv-900 catalyst.

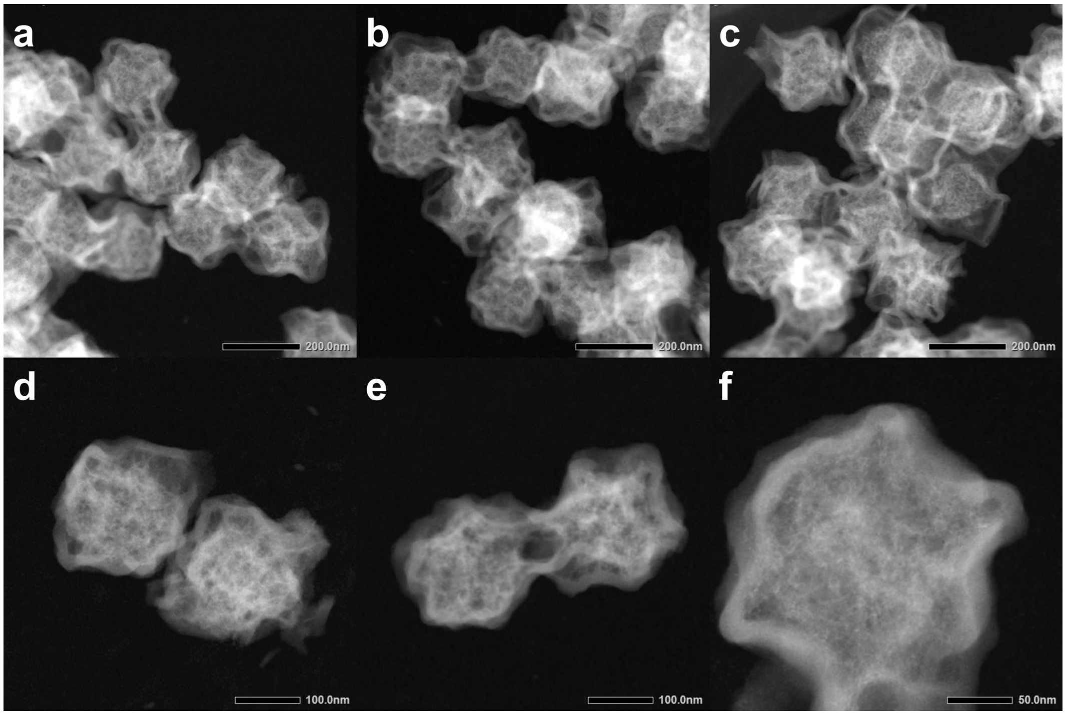

Supplementary Fig. 34. a-**f**, The HAADF-STEM images of NCv-900 catalyst from different regions at different magnifications. The NCv-900 also had abundant mesopores and micropores, the same as Fe-NCv-900 by self-carbon-thermal-reduction strategy.

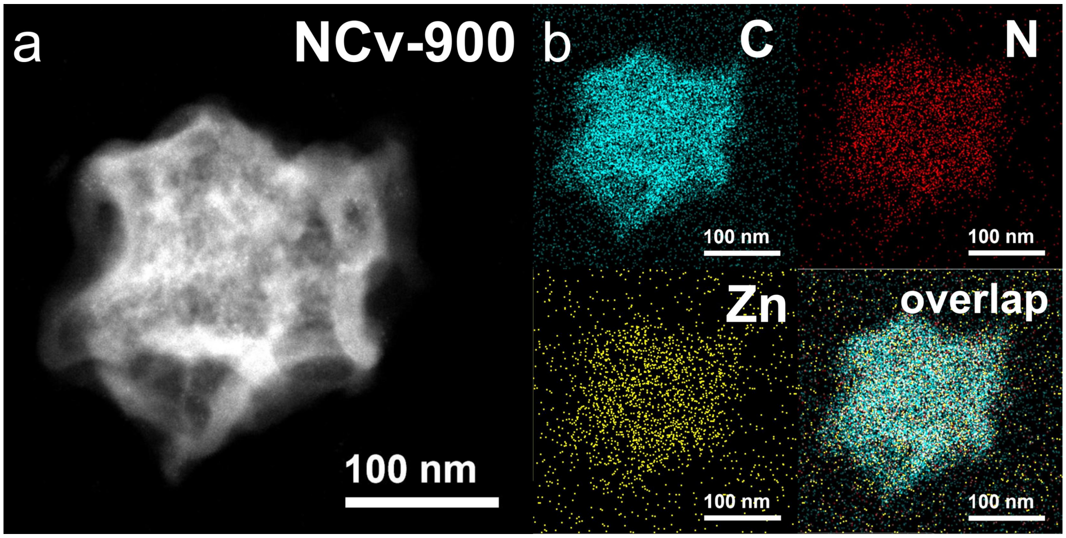

Supplementary Fig. 35. **a**, The HAADF-STEM image of NCv-900. **b**, The corresponding EDX spectroscopy elemental mapping results of NCv-900.

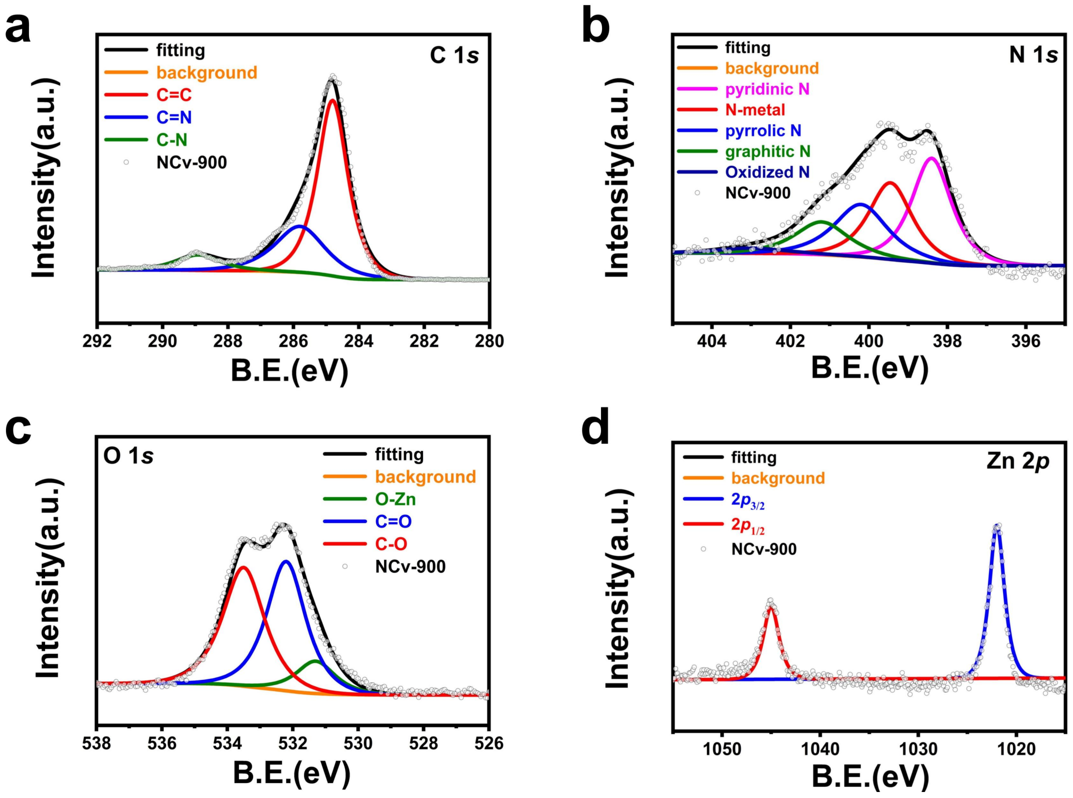

Supplementary Fig. 36. The XPS spectra of NCv-900. **a**, XPS spectrum for the C 1*s*. **b**, XPS spectrum for the N 1*s*. **c**, XPS spectrum for the O 1*s*. **d**, XPS spectrum for the Zn 2*p*.

For C 1*s* spectra, C=C, C=N and C-N bonds (*Angew. Chem. Int. Ed.* **59**, 22465-22469 (2020).) co-existed. For N 1*s* spectra, pyridinic N, N-metal, pyrrolic N, and graphitic N species (*Angew. Chem. Int. Ed.* **60**, 9078-9085 (2021).) co-existed. For O 1*s* spectra, C=O, C-O bonds (*Angew. Chem. Int. Ed.* **59**, 1961-1965 (2020).) and O-Zn bond (*Appl. Catal. B-Environ.* **310**, 121298 (2022).) co-existed.

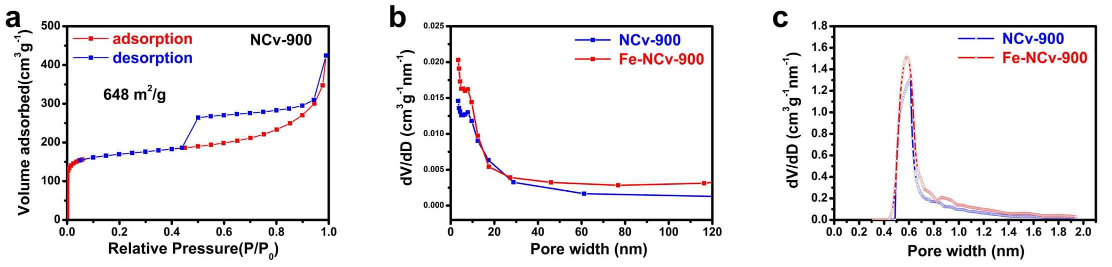

Supplementary Fig. 37. The BET surface area and pore size distribution of NCv-900. **a**, N_2 adsorption-desorption isotherms and corresponding BET surface area. **b**, The comparison of mesopore-size distribution of NCv-900 and Fe-NCv-900. **c**, The comparison of micropore-size distribution of NCv-900 and Fe-NCv-900.

The BET surface area of NCv-900 is 648 m^2/g. The NCv-900 catalyst also has abundant mesopores and micropores, the same as Fe-NCv-900 catalyst, measured by nitrogen sorption isotherm experiments.

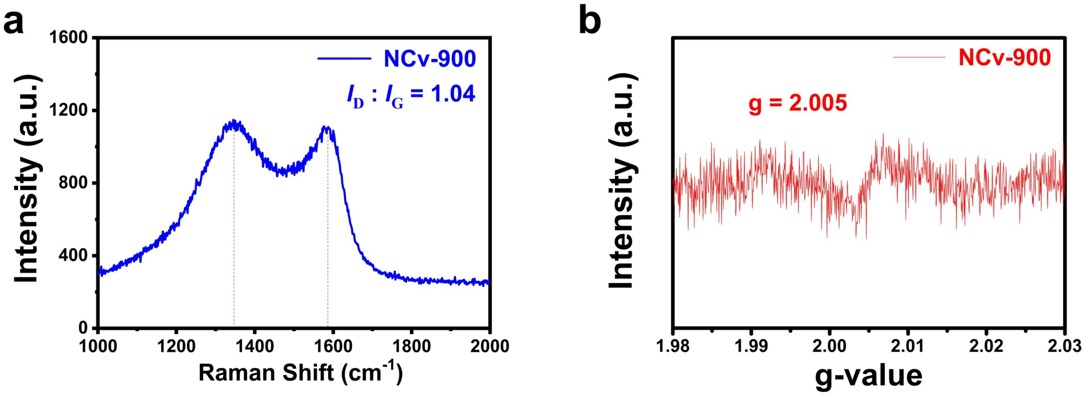

Supplementary Fig. 38. a, The Raman spectrum of NCv-900. **b**, The EPR spectrum of NCv-900.

The Raman spectrum of NCv-900 is shown in **Supplementary Fig. 38a**. The I_D/I_G value of NCv-900 catalyst is 1.04, similar to those of Fe-NCv-800 (I_D/I_G = 1.05) and Fe-NCv-900 (I_D/I_G = 1.07), indicating the existence of disorder structure of NCv-900. The EPR spectrum of NCv-900 is shown in **Supplementary Fig. 38b**, with an obvious signal at around 2.005 g, indicating that the existence of carbon-vacancies in the carbon substrate of NCv-900. (*Adv. Mater.* **35**, 2210714 (2023).)

Therefore, except for absence of Fe element, NCv-900 catalyst has the same characteristic with the Fe-NCv-900 catalyst, with the existence of Zn element, abundant mesopores, disorder structure and carbon-vacancies in the carbon substrate.

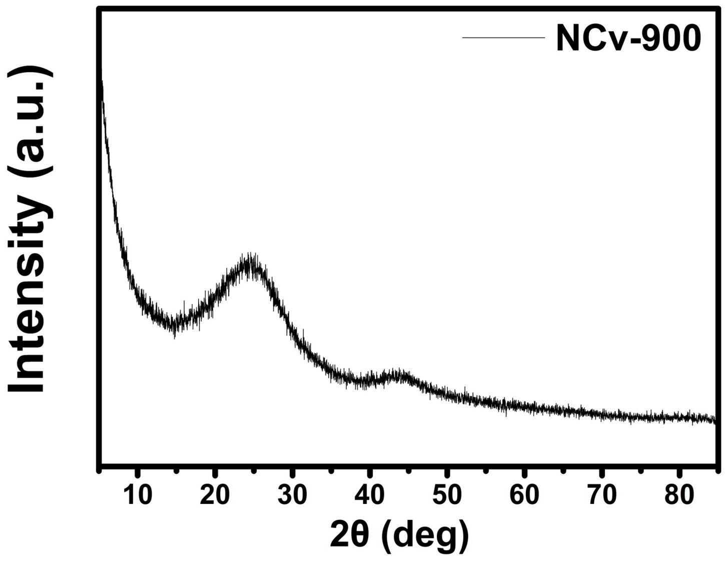

Supplementary Fig. 39. The XRD spectrum of NCv-900. The NCv-900 only had two broad peaks around 25° and 44°, which was ascribed to the characteristic carbon (002) and (100)/(101) diffractions, respectively.

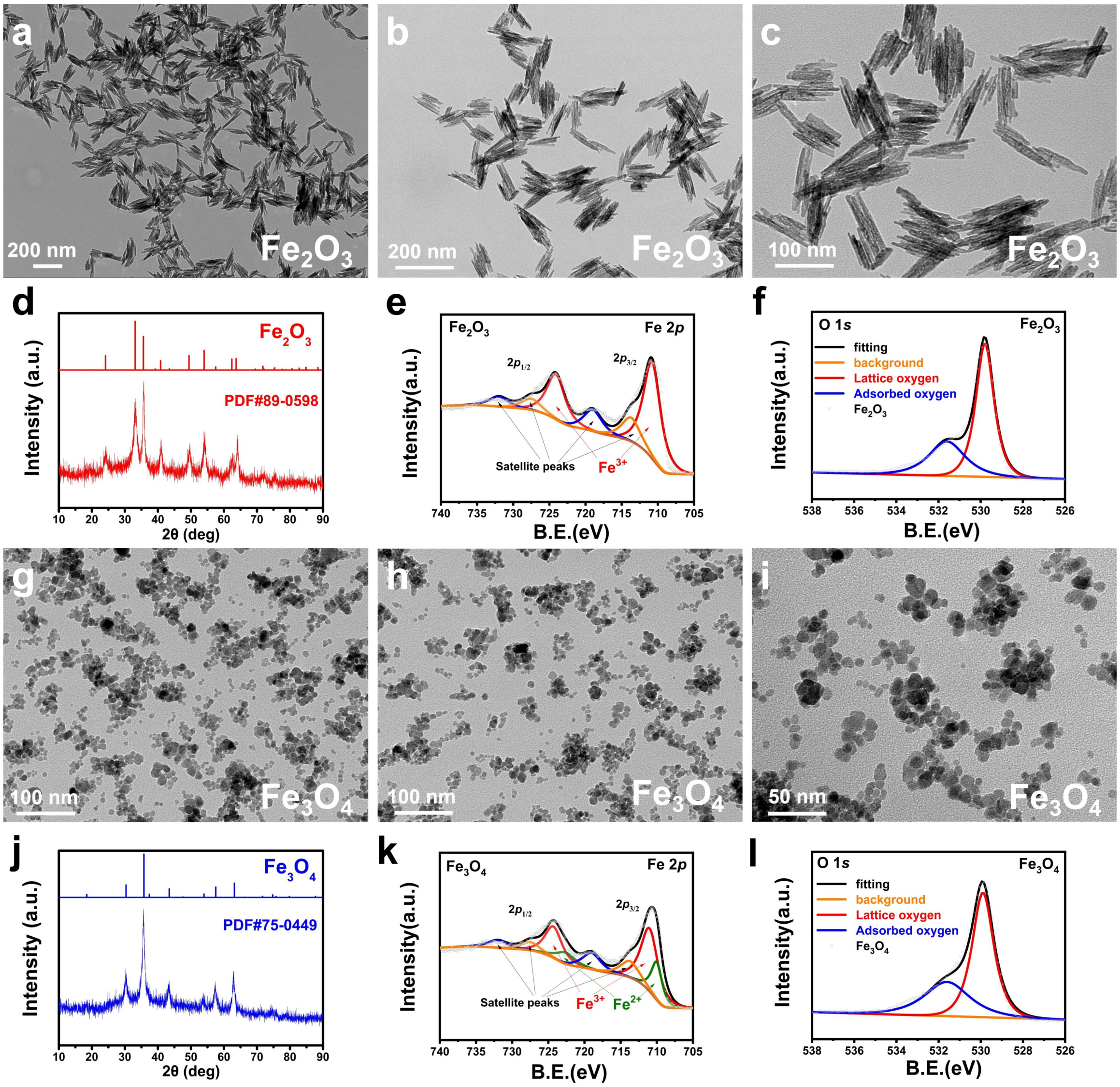

Supplementary Fig. 40. The characterizations of Fe_2O_3 and Fe_3O_4 nano-catalysts by TEM, XRD and XPS measurements. **a**-**c**, The TEM images of Fe_2O_3. **d**, The XRD pattern of Fe_2O_3. **e**, The XPS spectrum of Fe_2O_3 for the Fe 2*p*. **f**, The XPS spectrum of Fe_2O_3 for the O 1*s*. **g**-**i**, The TEM images of Fe_3O_4. **j**, The XRD pattern of Fe_3O_4. **k**, The XPS spectrum of Fe_3O_4 for the Fe 2*p*. **l**, The XPS spectrum of Fe_3O_4 for the O 1*s*.

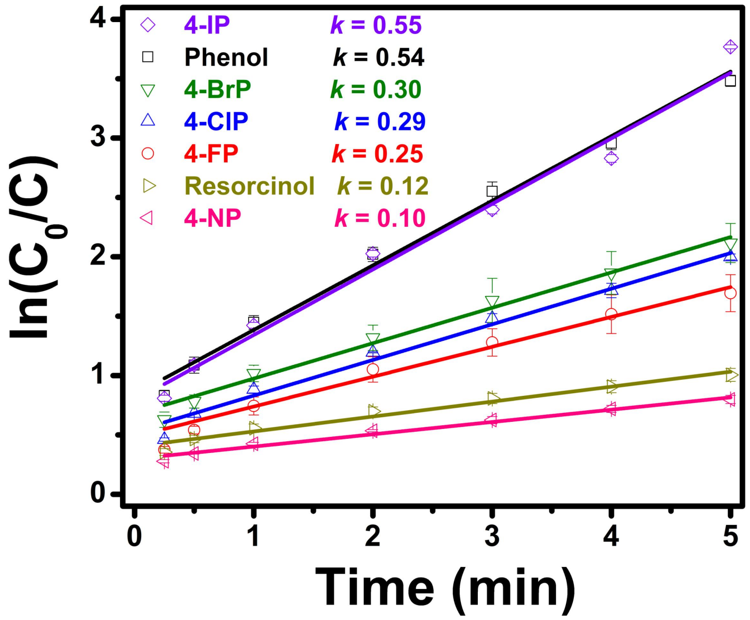

Supplementary Fig. 41. The comparison of the first-order kinetic constant *k* between 4-NP, resorcinol, 4-FP, 4-ClP, 4-BrP, 4-IP and phenol catalyzed by Fe-NCv-900.

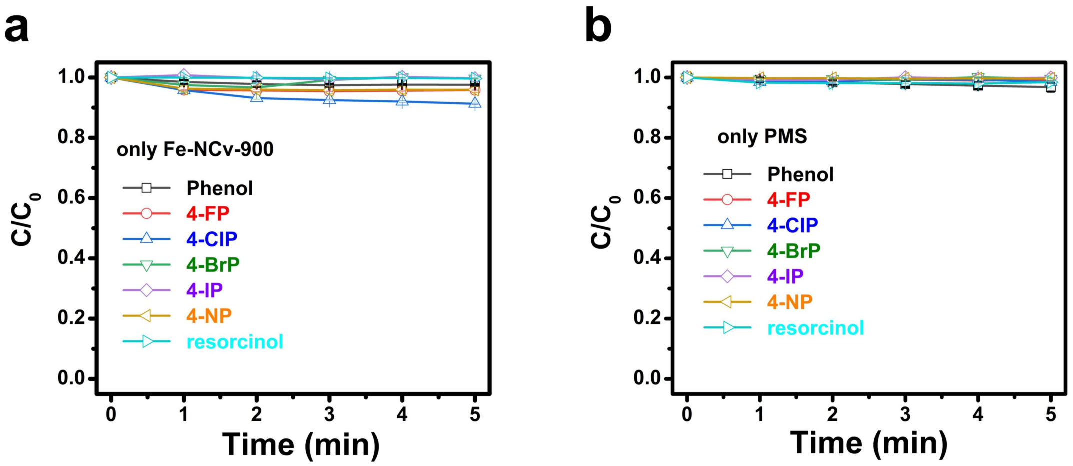

Supplementary Fig. 42. **a**, The adsorption of phenolic pollutants on Fe-NCv-900. ($[catalyst]_0$ = 10 mg/L, $[contaminants]_0$ = 10 μM, initial pH = 7.0±0.1) **b**, The direct degradation by oxidation of PMS. ($[contaminants]_0$ = 10 μM, $[PMS]_0$ = 100 μM, initial pH = 7.0±0.1)

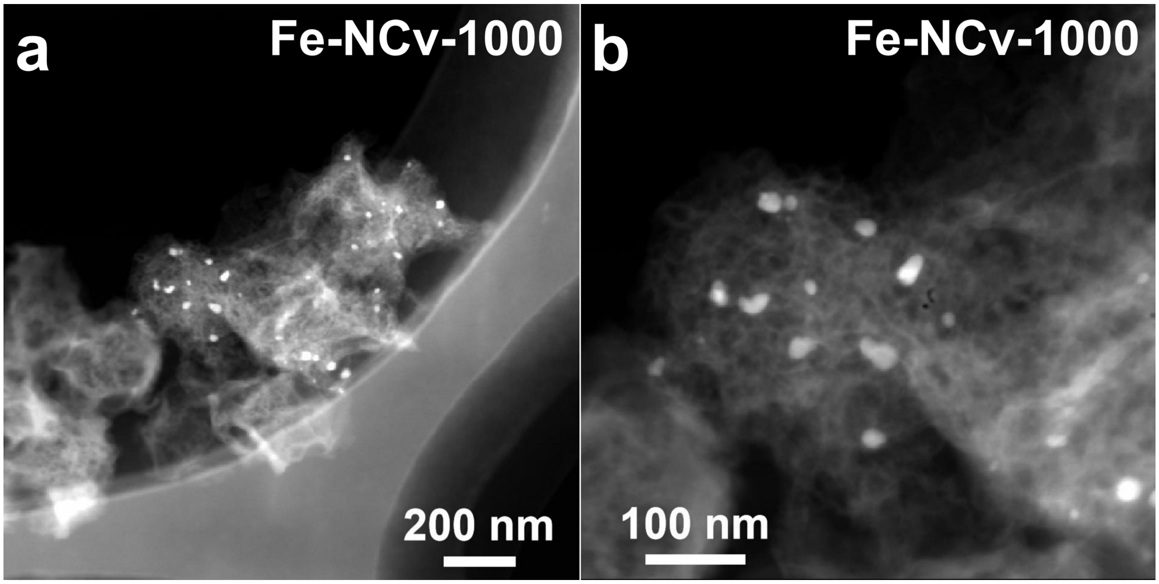

Supplementary Fig. 43. a-b, The HAADF-STEM images of the Fe-NCv-1000 catalyst from different regions at different magnifications. The partial Fe nanoparticles formed in the Fe-NCv-1000 catalyst. Therefore, the Fe-NCv-1000 catalyst had lower atomic utilization efficiency during catalysis due to the formation of partial Fe nanoparticles.

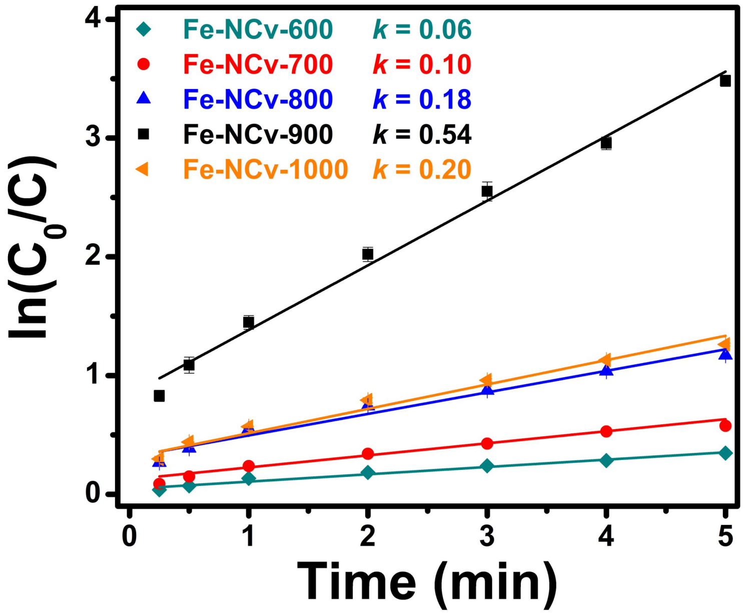

Supplementary Fig. 44. The comparison of the first-order kinetic constant *k* for degradation of phenol between different Fe-NCv catalysts under pyrolysis at different temperatures.

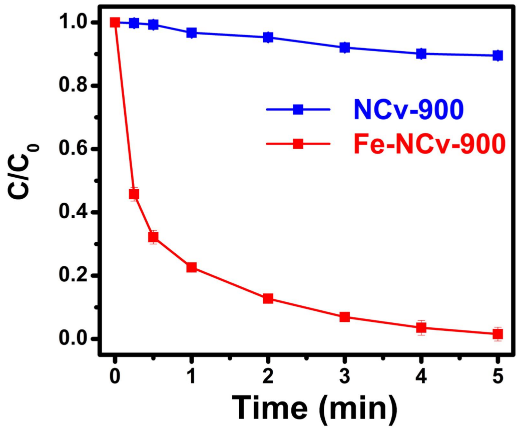

Supplementary Fig. 45. The plots of phenol concentration versus time of Fe-NCv-900 and NCv-900 catalysts.

For degradation of phenol by Fenton-like reaction, Fe-NCv-900 catalyst exhibits much higher catalytic activity compared to NCv-900 catalyst without Fe loading. As shown in **Supplementary Fig. 45**, during degradation of phenol for 5 min, the removal ratios of phenol catalyzed by Fe-NCv-900 and NCv-900 are 97% and 10%, respectively, indicating the Fe ISAS is the catalytic site in Fe-NCv-900 catalyst during catalysis.

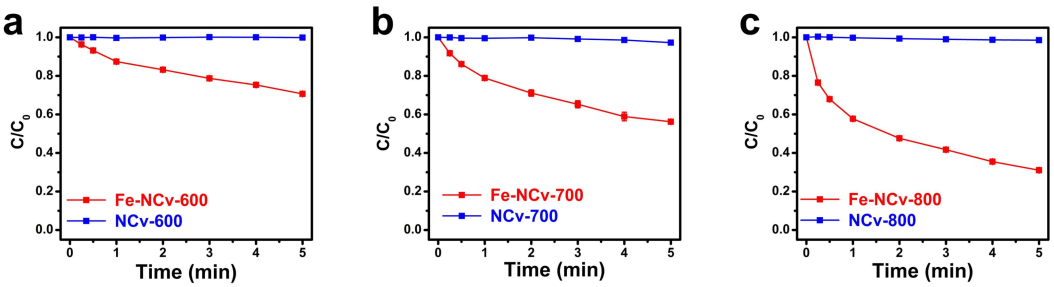

Supplementary Fig. 46. a, The plots of phenol concentration versus time of Fe-NCv-600 and NCv-600 catalysts. **b**, The plots of phenol concentration versus time of Fe-NCv-700 and NCv-700 catalysts. **c**, The plots of phenol concentration versus time of Fe-NCv-800 and NCv-800 catalysts.

Similarly, we also synthesize NCv-800, NCv-700 and NCv-600 without Fe loading by pyrolysis of pure ZIF-8 and filter papers without $Fe(acac)_3$ loading at 800°C, 700°C and 600°C, respectively. As shown in **Supplementary Fig. 46**, compared with Fe-NCv-800, Fe-NCv-700 and Fe-NCv-600, the catalytic activities of NCv-800, NCv-700 and NCv-600 are almost inert, which well exclude the impacts of Zn element during catalysis.

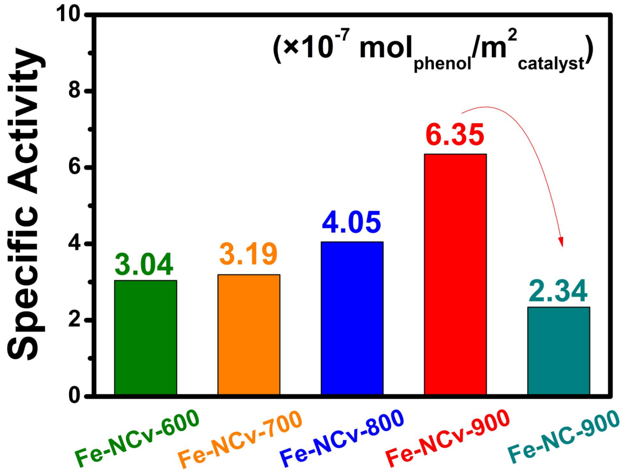

Supplementary Fig. 47. The comparison of specific activities ($mol_{phenol}/m^2_{catalyst}$) between different Fe-NC catalysts.

In order to give a fair comparison of the activities of Fe-NCv catalysts by pyrolysis at different temperatures, we compare the activities of Fe-NCv catalysts at lower conversion of phenol (the removal ratios of phenol after degradation for 0.25 min), because the low concentration of phenol in solution at higher conversion will affect the reaction rates.

As shown in **Supplementary Fig. 47**, the specific activities ($mol_{phenol}/m^2_{catalyst}$) of Fe-NCv catalysts increase gradually from Fe-NCv-600 to Fe-NCv-900, which are higher than that of Fe-NC-900 without abundant carbon-defect. Therefore, the increasing BET surface area is not the primary factor for the increasing activities of Fe-NCv catalysts.

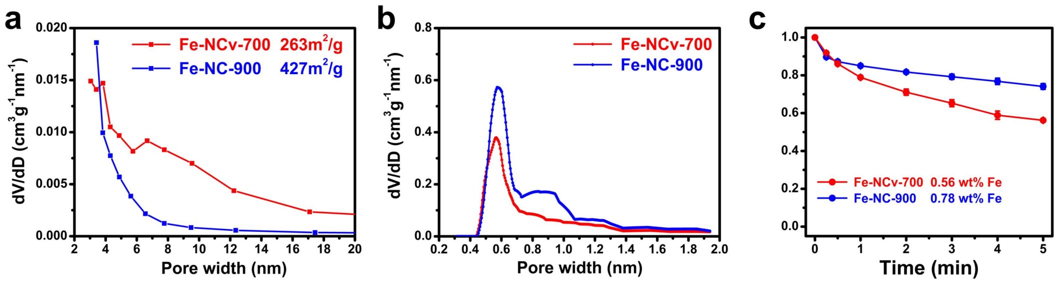

Supplementary Fig. 48. The comparison of distribution of **a** mesopores, **b** micropores and **c** catalytic activity between Fe-NCv-700 and Fe-NC-900.

In order to illustrate the effect of pores on catalytic activity, we compare the distribution of pore's size and catalytic activities of Fe-NCv-700 and Fe-NC-900 catalysts. As shown in **Supplementary Fig. 48**, Fe-NCv-700 has more mesopores while Fe-NC-900 has more micropores (**Supplementary Fig. 48a** and **b**). Considering the molecular diameter of phenol is around 0.69 nm and mesopores will not hinder the diffusion of phenol, therefore more micropores are advantageous for the diffusion of phenol and catalysis. Besides, the BET surface area of Fe-NC-900 is 427 m^2/g, higher than that of Fe-NCv-700 with the BET surface area of 263 m^2/g. The Fe loading of Fe-NC-900 is 0.78 wt% Fe, also higher than that of Fe-NCv-700 with 0.56 wt% Fe. Therefore, compared with Fe-NCv-700, the more micropores, larger BET surface area and higher Fe loading of Fe-NC-900 are more advantageous for boosting catalytic activity. However, for degradation of phenol by Fenton-like reaction, Fe-NCv-700 exhibits higher activity than that of Fe-NC-900, as shown in **Supplementary Fig. 48c**, indicating that the structure of pores and the BET surface area are not the major factor during catalysis.

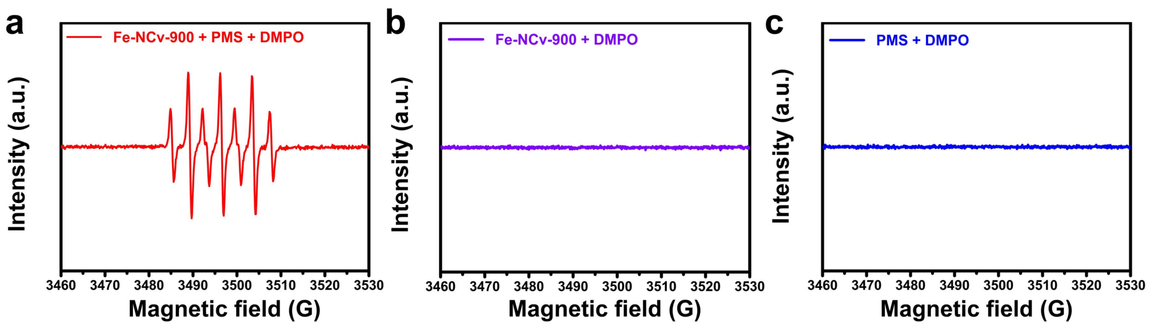

Supplementary Fig. 49. The EPR experiments with 5,5-dimethyl-1-pyrroline N-oxide (DMPO) as a trapping agent. **a**, Fe-NCv-900/PMS system ([catalyst]$_0$ = 10 mg/L, [DMPO]$_0$ = 100 mM, [PMS]$_0$ = 100 μM, initial pH = 7.0±0.1). **b**, only Fe-NCv-900 ([catalyst]$_0$ = 10 mg/L, [DMPO]$_0$ = 100 mM, initial pH = 7.0±0.1). **c**, only PMS ([DMPO]$_0$ = 100 mM, [PMS]$_0$ = 100 μM, initial pH = 7.0±0.1).

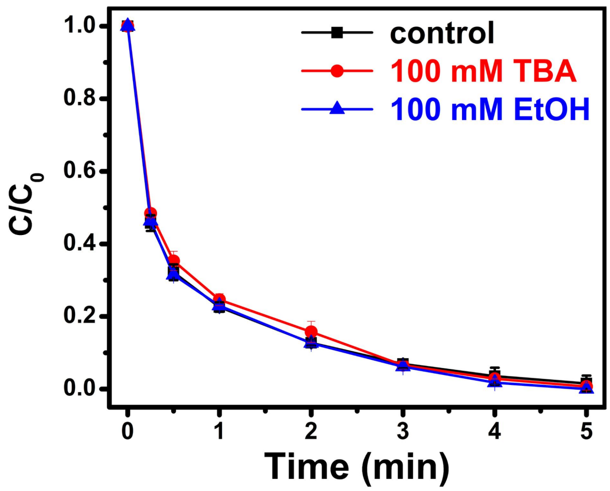

Supplementary Fig. 50. The quenching experiments with TBA or EtOH as quenching agents to scavenge $HO^{\bullet}$ and $SO_4^{\bullet-}$ radicals. ($[catalyst]_0$ = 10 mg/L, $[Phenol]_0$ = 10 μM, $[PMS]_0$ = 100 μM, initial pH = 7.0±0.1)

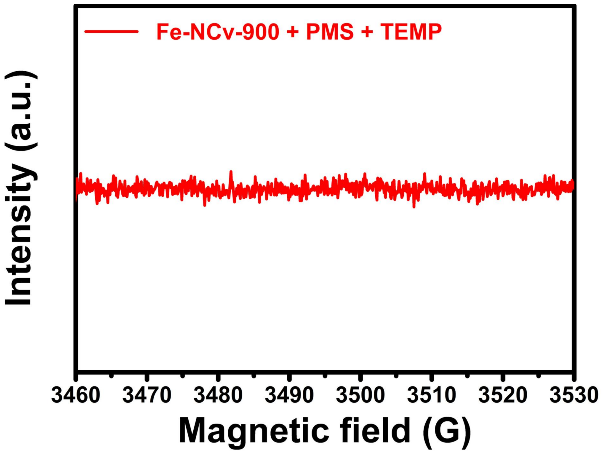

Supplementary Fig. 51. The EPR spectrum with TEMP as spin trapping agents for singlet oxygen ($^1O_2$). ($[catalyst]_0$ = 10 mg/L, $[TEMP]_0$ = 100 mM, $[PMS]_0$ = 100 μM, initial pH = 7.0 ± 0.1)

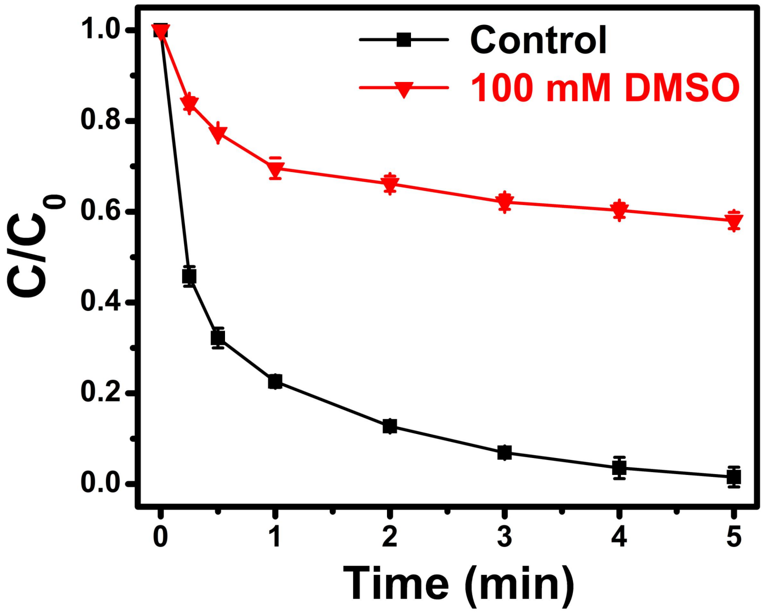

Supplementary Fig. 52. The inhibition experiment of the high-valent iron-oxo species by adding 100 mM Dimethyl sulfoxide (DMSO) as the inhibitor of high-valent iron-oxo species in Fe-NCv-900/PMS system for degradation of phenol.

Therefore, additional inhibition experiment is performed to confirm the high-valent iron-oxo species as active sites. We add 100 mM Dimethyl sulfoxide (DMSO) as the inhibitor of high-valent iron-oxo species in Fe-NCv-900/PMS system for degradation of phenol. As reported by the reference (*Angew. Chem.* **117**, 7031-7034 (2005).), the DMSO can consume the high-valent iron-oxo species by oxygen-atom-transfer step.

As shown in **Supplementary Fig. 52**, after adding 100 mM Dimethyl sulfoxide (DMSO) as the inhibitor of high-valent iron-oxo species, the removal ratio of phenol is 42.0% after 5 min for degradation, with an obvious decline compared to the control group without DMSO (97% removal ratio of phenol after 5 min for degradation), indicating that the decreasing activity of Fe-NCv-900 is attributed to the inhibition of DMSO for high-valent iron-oxo species, which is consistent with previous studies (*Appl. Catal. B: Environ.* **305**, 123049 (2022); *Chem. Eng. J.* **427**, 130803 (2022).).

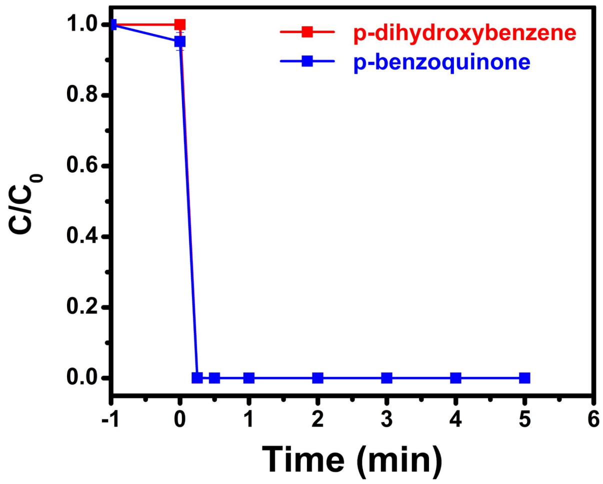

Supplementary Fig. 53. The degradation of p-dihydroxybenzene and p-benzoquinone by Fenton-like reaction. ($[catalyst]_0$ = 10 mg/L, $[contaminants]_0$ = 10 μM, $[PMS]_0$ = 100 μM, initial pH = 7.0 ± 0.1)

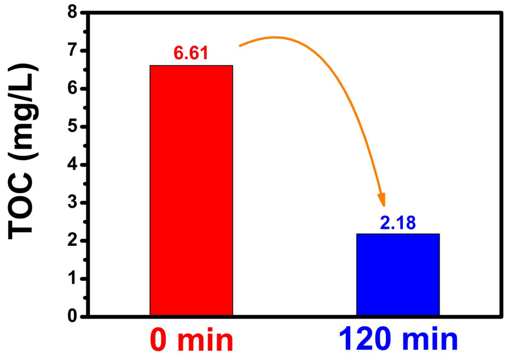

Supplementary Fig. 54. The total organic carbon (TOC) values for degradation of phenol at 0 min and 120min. ([catalyst]$_0$ = 30 mg/L, [contaminants]$_0$ = 100 μM, [PMS]$_0$ = 300 μM, initial pH = 7.0 ± 0.1)

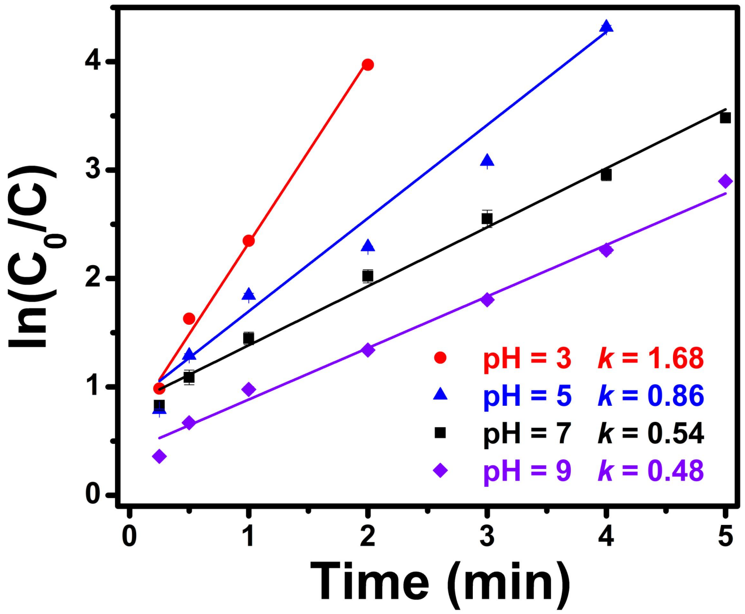

Supplementary Fig. 55. The comparison of the first-order kinetic constant *k* for degradation of phenol catalyzed by Fe-NCv-900 under different pH values.

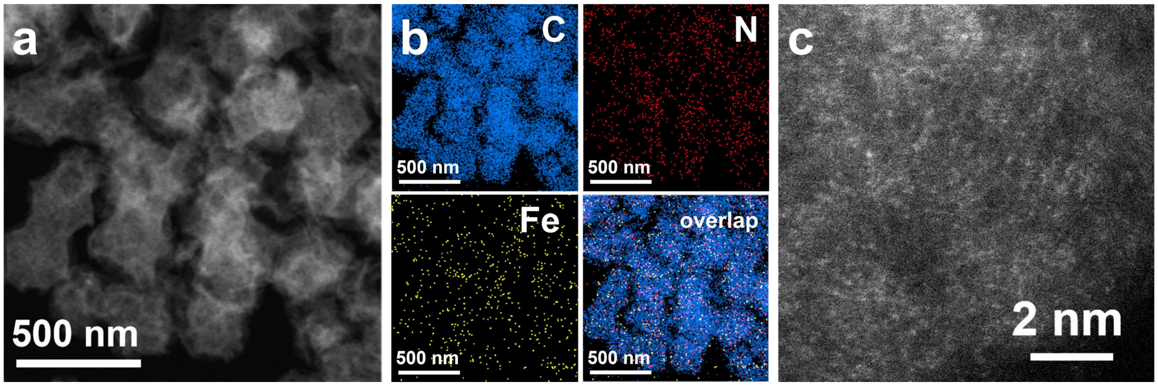

Supplementary Fig. 56. a, The HAADF-STEM image of Fe-NCv-900 after degradation of phenol by Fenton-like reaction. **b**, The corresponding EDX spectroscopy elemental mapping results. **c**, The corresponding AC-STEM image.

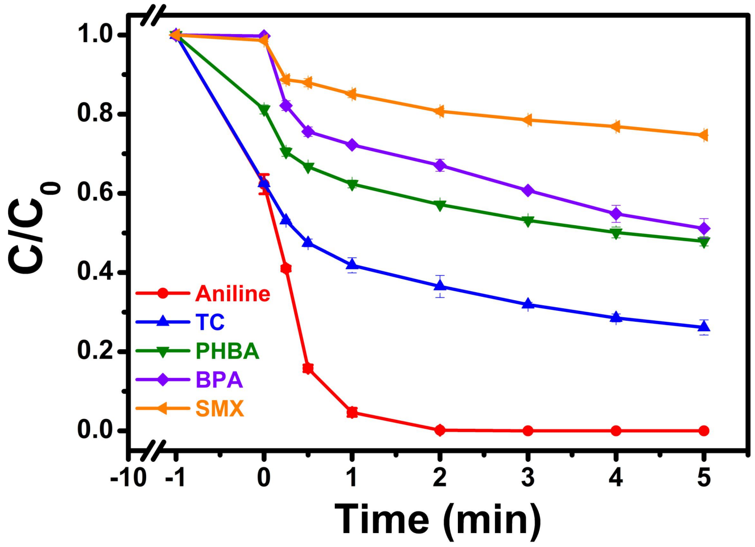

Supplementary Fig. 57. Degradation of aniline, Tetracycline (TC), p-Hydroxybenzoic acid (PHBA), bisphenol A (BPA), and sulfamethoxazole (SMX).

We studied the degradation of other organic pollutants by Fe-NCv-900/PMS system, such as aniline, Tetracycline (TC), p-Hydroxybenzoic acid (PHBA), bisphenol A (BPA), and sulfamethoxazole (SMX). The removal ratios of aniline, TC, PHBA, BPA, and SMX after degradation for 5 minutes were 100.0%, 73.9%, 52.1%, 48.9%, 25.3%, respectively, indicating that the high-valent iron-oxo species had different selectivity for degradation of different organic pollutants in Fe-NCv-900/PMS system, which was consistent with previous studies (*Environ. Sci. Technol.* **55**, 7034-7043 (2021); *Environ. Sci. Technol.* **54**, 14057-14065 (2020); *Environ. Sci. Technol.* **52**, 2197-2205 (2018).).

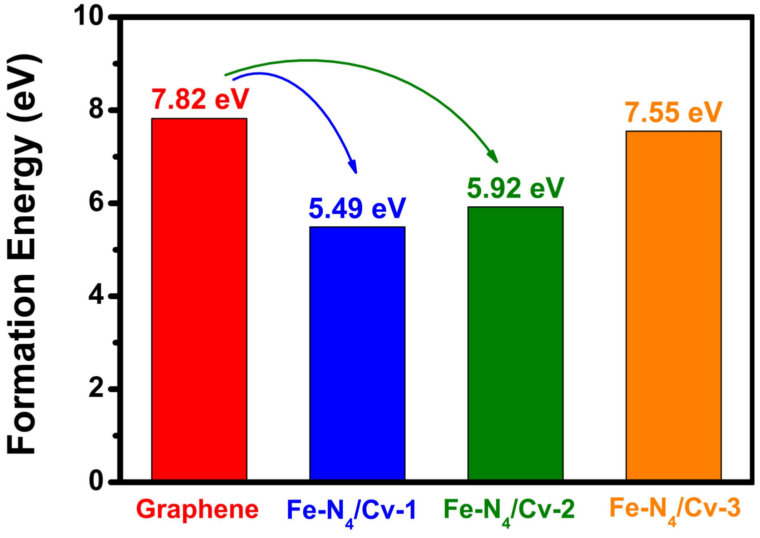

Supplementary Fig. 58. The formation energy of carbon-atom vacancy on pristine graphene and Fe-N_4-sites-doped graphene.

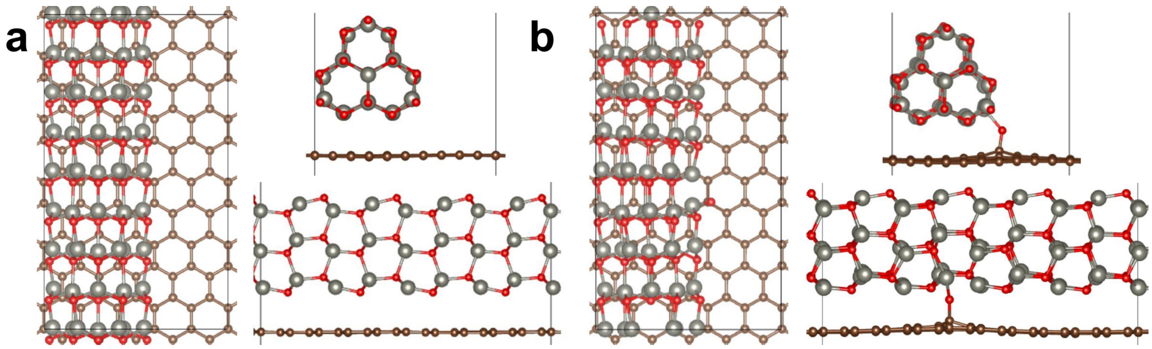

Supplementary Fig. 59. a-b, The top view and side view of the optimized structure of ZnO@graphene hetero-structure before and after C-O bonding.

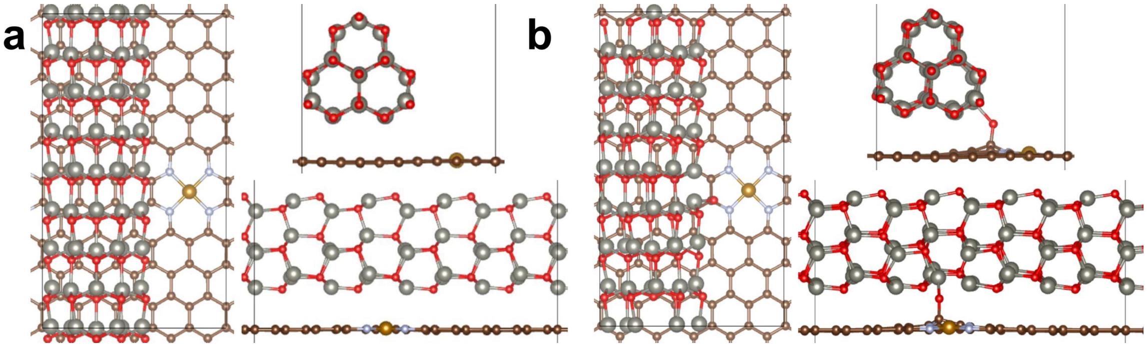

Supplementary Fig. 60. a-b, The top view and side view of the optimized structure of ZnO@Fe-N_4/N-doped graphene hetero-structure for synthesis of Fe-N_4/Cv-1 before and after C-O bonding.

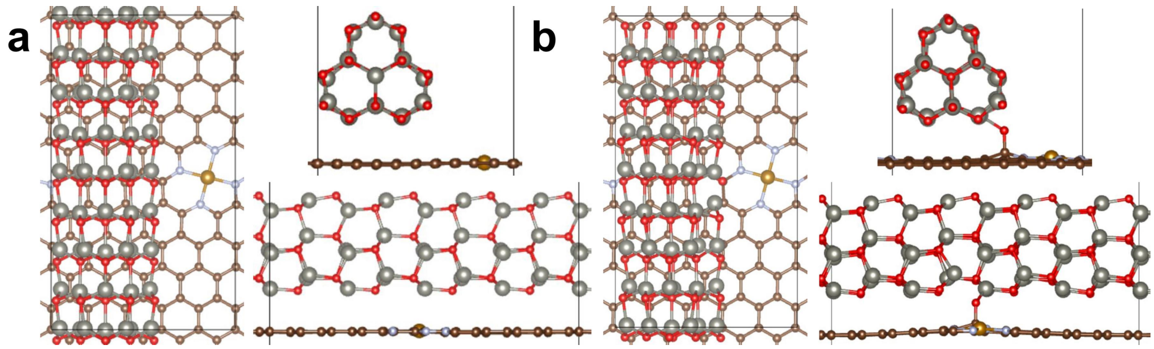

Supplementary Fig. 61. a-b, The top view and side view of the optimized structure of ZnO@Fe-N_4/N-doped graphene hetero-structure for synthesis of Fe-N_4/Cv-2 before and after C-O bonding.

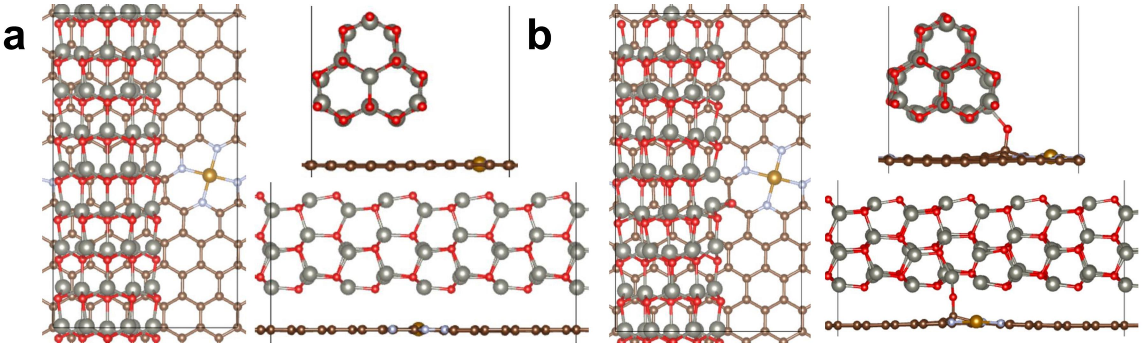

Supplementary Fig. 62. a-b, The top view and side view of the optimized structure of ZnO@Fe-N_4/N-doped graphene hetero-structure for synthesis of Fe-N_4/Cv-3 before and after C-O bonding.

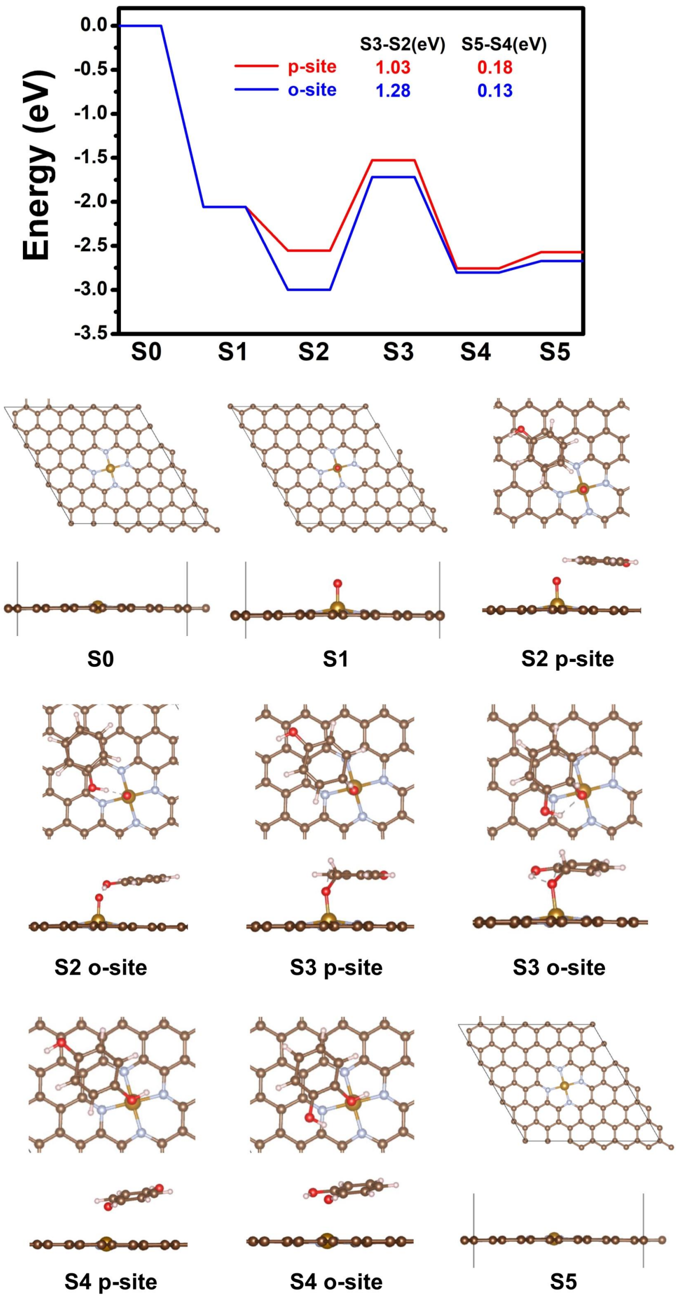

Supplementary Fig. 63. The catalytic pathway for degradation of phenol on ortho position (o-site) and para position of hydroxyl group (p-site) of phenol catalyzed by Fe-N_4/N-doped graphene and corresponding structures.

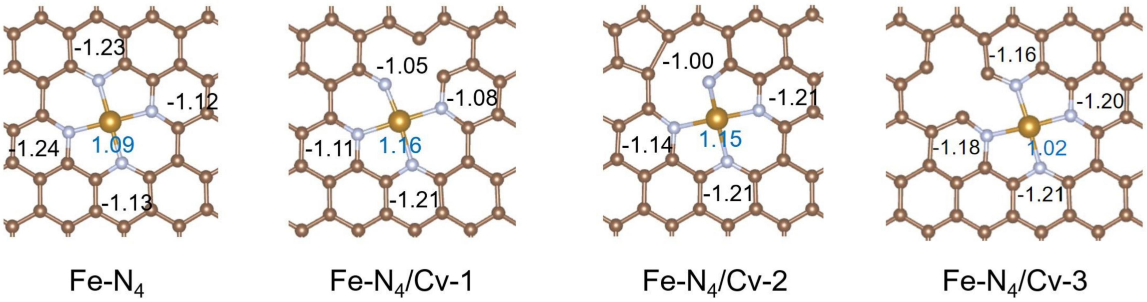

Supplementary Fig. 64. The analysis of net Bader charge of Fe-N_4, Fe-N_4/Cv-1, Fe-N_4/Cv-2 and Fe-N_4/Cv-3 sites on N-doped graphene. The brown, blue, and dark yellow balls represented C, N, and Fe atoms, respectively. The values in black and blue represented the net Bader charges of N and Fe atoms, respectively.

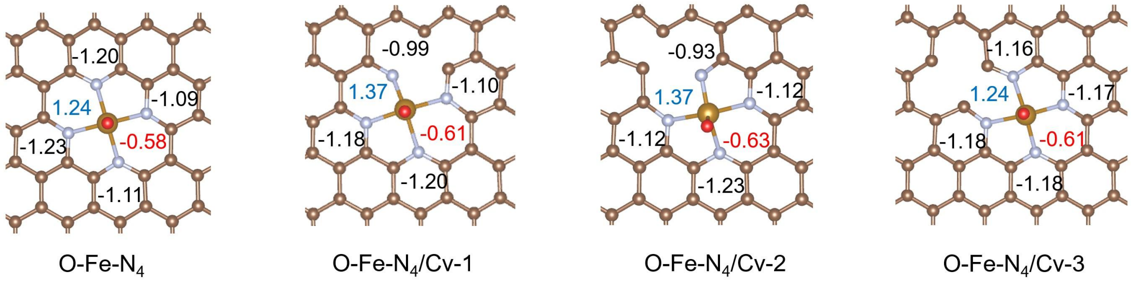

Supplementary Fig. 65. The analysis of net Bader charge of O-Fe-N_4, O-Fe-N_4/Cv-1, O-Fe-N_4/Cv-2 and O-Fe-N_4/Cv-3 sites on N-doped graphene. The brown, blue, red and dark yellow balls represented C, N, O and Fe atoms, respectively. The values in black, blue and red represented the net Bader charges of N, Fe and O atoms, respectively.

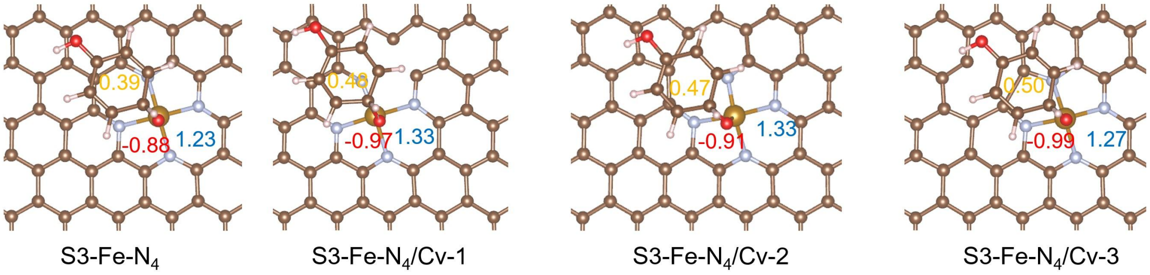

Supplementary Fig. 66. The analysis of net Bader charge of S3-Fe-N_4, S3-Fe-N_4/Cv-1, S3-Fe-N_4/Cv-2 and S3-Fe-N_4/Cv-3 sites on N-doped graphene. The brown, blue, red and dark yellow balls represented C, N, O and Fe atoms, respectively. The values in blue, red and yellow represented the net Bader charges of Fe, O and C atoms, respectively.

Supplementary Table 1. The Fe and Zn contents of Fe-NCv catalysts by self-carbon-thermal-reduction measured by ICP-OES measurement.

**Catalyst**	**Fe loading (wt%)**	**Zn loading (wt%)**
Fe-NCv-400	0.32	28.73
Fe-NCv-500	0.34	27.53
Fe-NCv-600	0.63	26.31
Fe-NCv-700	0.56	19.87
Fe-NCv-800	0.83	9.89
Fe-NCv-900	0.75	1.67
Fe-NC-900	0.78	9.74
ZIF-8	-	27.20

Supplementary Table 2. The structural parameters extracted from the Fe K-edge EXAFS fitting.

Sample	Shell	CN	R(Å)	σ^2(Å^2)	ΔE_0(eV)	R factor
Fe foil	Fe-Fe[1]	8*	2.45	0.0066	1.443	0.003
	Fe-Fe[2]	6*	2.83	0.0034	1.443	
Fe-NCv-900	Fe-N	4.1	1.98	0.0107	7.187	0.001
Fe-NC-900	Fe-N	4.0	1.99	0.0111	8.768	0.009

CN was coordination numbers. R was bond distance. σ^2 was Debye-Waller factors. ΔE_0 was the inner potential correction. R factor reflected goodness of fitting. S_0^2 was set to 0.83, according to the experimental EXAFS fitting of Fe foil by fixing CN as the known crystallographic value. Error bounds were estimated as CN ± 20%; R ± 1%; σ^2 ± 20%.

Supplementary Table 3. The structural parameters of Fe-N_4, Fe-N_4/Cv-1, Fe-N_4/Cv-2 and Fe-N_4/Cv-3 sites on N-doped graphene by DFT calculation.

**Catalytic Sites**	**d_{min} (Fe-N)**	**net Bader Charge (Fe)**	**d band center**
Fe-N_4	1.89 Å	1.09 |e|	-0.94 eV
Fe-N_4/Cv-1	1.72 Å	1.16 |e|	-1.45 eV
Fe-N_4/Cv-2	1.77 Å	1.15 |e|	-1.23 eV
Fe-N_4/Cv-3	1.85 Å	1.02 |e|	-0.89 eV

Supplementary Table 4. The structural parameters of O-Fe-N_4, O-Fe-N_4/Cv-1, O-Fe-N_4/Cv-2 and O-Fe-N_4/Cv-3 sites on N-doped graphene by DFT calculation.

**Catalytic Sites**	**d (Fe-O)**	**net Bader Charge (Fe)**	**net Bader Charge (O)**	**ICOHP_total (Fe-N)**	**ICOHP (Fe-O)**
O-Fe-N_4	1.65 Å	1.24 |e|	-0.58 |e|	-2.38 eV	-5.74 eV
O-Fe-N_4/Cv-1	1.66 Å	1.37 |e|	-0.61 |e|	-4.22 eV	-5.70 eV
O-Fe-N_4/Cv-2	1.66 Å	1.37 |e|	-0.63 |e|	-4.19 eV	-5.67 eV
O-Fe-N_4/Cv-3	1.65 Å	1.24 |e|	-0.61 |e|	-1.96 eV	-5.64 eV

Supplementary Table 5. The structural parameters of S3-Fe-N_4, S3-Fe-N_4/Cv-1, S3-Fe-N_4/Cv-2 and S3-Fe-N_4/Cv-3 sites on N-doped graphene by DFT calculation.

**Catalytic Sites**	**d (Fe-O)**	**ICOHP (Fe-O)**	**d (phenol C-O)**	**ICOHP (phenol C-O)**
S3-Fe-N_4	1.83 Å	-2.99 eV	1.46 Å	-9.48 eV
S3-Fe-N_4/Cv-1	1.91 Å	-2.24 eV	1.41 Å	-10.36 eV
S3-Fe-N_4/Cv-2	1.83 Å	-2.93 eV	1.45 Å	-9.60 eV
S3-Fe-N_4/Cv-3	1.85 Å	-2.70 eV	1.43 Å	-10.05 eV
